# Zinc acquisition and its contribution to *Klebsiella pneumoniae* virulence

**DOI:** 10.3389/fcimb.2023.1322973

**Published:** 2024-01-05

**Authors:** Eve A. Maunders, Matthew W. Giles, Katherine Ganio, Bliss A. Cunningham, Vicki Bennett-Wood, Gregory B. Cole, Dixon Ng, Christine C. Lai, Stephanie L. Neville, Trevor F. Moraes, Christopher A. McDevitt, Aimee Tan

**Affiliations:** ^1^ Department of Microbiology and Immunology, The Peter Doherty Institute for Infection and Immunity, University of Melbourne, Melbourne, VIC, Australia; ^2^ Department of Biochemistry, University of Toronto, Toronto, ON, Canada

**Keywords:** *Klebsiella pneumoniae*, zinc homeostasis, periplasmic binding protein, ABC transporter, zinc transport, bacterial virulence

## Abstract

*Klebsiella pneumoniae* is a World Health Organization priority pathogen and a significant clinical concern for infections of the respiratory and urinary tracts due to widespread and increasing resistance to antimicrobials. In the absence of a vaccine, there is an urgent need to identify novel targets for therapeutic development. Bacterial pathogens, including *K. pneumoniae*, require the *d*-block metal ion zinc as an essential micronutrient, which serves as a cofactor for ~6% of the proteome. During infection, zinc acquisition necessitates the use of high affinity uptake systems to overcome niche-specific zinc limitation and host-mediated nutritional immunity. Here, we report the identification of ZnuCBA and ZniCBA, two ATP-binding cassette permeases that are highly conserved in *Klebsiella* species and contribute to *K. pneumoniae* AJ218 zinc homeostasis, and the high-resolution structure of the zinc-recruiting solute-binding protein ZniA. The Znu and Zni permeases appear functionally redundant with abrogation of both systems required to reduce *K. pneumoniae* zinc accumulation. Disruption of both systems also exerted pleiotropic effects on the homeostasis of other *d*-block elements. Zinc limitation perturbed *K. pneumoniae* cell morphology and compromised resistance to stressors, such as salt and oxidative stress. The mutant strain lacking both systems showed significantly impaired virulence in acute lung infection models, highlighting the necessity of zinc acquisition in the virulence and pathogenicity of *K. pneumoniae*.

## Introduction

*Klebsiella pneumoniae* is a Gram-negative bacterial pathogen of global priority, due to rising rates of antimicrobial resistance in clinical settings (Wyres et al., 2020). Although primarily considered to be a healthcare-associated infection of the respiratory and urinary tracts, there are increasing reports of hypervirulent community-acquired isolates with more severe disease presentations, such as liver abscesses, bloodstream infections, and sepsis (Russo and Marr, 2019; Choby et al., 2020). While these distinct presentations are typically caused by different *K. pneumoniae* lineages, there are increasing reports of resistance determinants mobilizing into hypervirulent strains (Choby et al., 2020). In the absence of a vaccine, there is an urgent need to develop new antimicrobial strategies for this pathogen.

During colonization and infection, bacterial pathogens must acquire their essential nutrients, such as trace elements, from the host. To impair the ability of invading pathogens to scavenge nutrients, vertebrate hosts have evolved complex strategies to limit their bioavailability at the host-pathogen interface. Withholding of first-row *d*-block elements, also called nutritional immunity, is frequently employed to render the metal ions manganese, iron, and zinc, poorly accessible to invading pathogens. Depriving pathogens of these elements prevents their incorporation into proteins as structural and/or catalytic centers, thereby limiting bacterial infection (Murdoch and Skaar, 2022). To date, studies of *K. pneumoniae* and metal homeostasis have predominantly focused on iron due to its frequent contribution to bacterial virulence and, in particular, its association with hypervirulent *K. pneumoniae* lineages that can encode up to four iron scavenging siderophore systems (Paczosa and Mecsas, 2016). In contrast, the role of other *d*-block elements in *K. pneumoniae* have remained less defined. Zinc, which commonly occurs as the divalent cation Zn(II), is the second most abundant *d*-block element in *K. pneumoniae* (Maunders et al., 2022). Recent studies of Zn(II)-limited *K. pneumoniae* K52145 suggested that metal limitation may influence histidine utilization and expression of the regulatory protein ChaB (Sukumaran et al., 2021). However, a systematic investigation of Zn(II) homeostasis in *K. pneumoniae* and identification of pathways involved in metal acquisition have not been performed.

In the Enterobacteriaceae family, which includes *K. pneumoniae*, numerous pathways have been associated with Zn(II) uptake (Porcheron et al., 2013). These include low affinity transporters such as ZupT, which facilitates Zn(II) uptake in *E. coli* when the metal is abundant (Grass et al., 2002; Grass et al., 2005); and divalent cation importers such as PitA, which has been associated with import of Zn(II) and other divalent *d*-block elements in *E. coli* (Beard et al., 2000). High affinity Zn(II) transporters are more frequently implicated as virulence factors due to their essentiality in overcoming host metal withholding mechanisms (Rosadini et al., 2011; Corbett et al., 2012; Plumptre et al., 2014; Sheng et al., 2015). In prokaryotes, the ATP-binding cassette (ABC) permeases are the most prevalent high affinity import pathways of Zn(II). In the Enterobacteriaceae, the prototypical example of this pathway is ZnuCBA from *E. coli* (Patzer and Hantke, 1998; Ammendola et al., 2007). This system is comprised of a cluster A-I solute-binding protein (SBP) that recruits Zn(II) from the periplasm, ZnuA, and an ABC transporter, ZnuBC, that actively translocates SBP-delivered metal ions into the cytosol. In *Salmonella enterica* serovar Typhimurium, the ZnuCBA system also interacts with ZinT, a periplasmic accessory protein that serves as a Zn(II)-metallochaperone (Petrarca et al., 2010).

Here, the Zn(II) acquisition pathways of *K. pneumoniae* strain AJ218 were investigated. This work identified ZnuCBA, an ortholog of the *E. coli* ZnuCBA permease, and a novel second ABC permease termed ZniCBA for its role in Zn(II) import. The ZniCBA permease is highly conserved in *Klebsiella* species and closely related Enterobacteriaceae, but is absent from other species such as *E. coli* and *S. enterica*. The ZnuCBA and ZniCBA permeases appear functionally redundant for Zn(II) uptake, with either system sufficient for wild-type growth and Zn(II) acquisition. However, deletion of both SBPs, ZnuA and ZniA, substantially impaired bacterial Zn(II) uptake by ~90% during growth in Zn(II)-limited media. Reduced cellular Zn(II) had pleiotropic impacts on cell morphology, stress resistance pathways, and reduced *K. pneumoniae* virulence in a murine model of acute lung infection. These findings provide new insights into how *K. pneumoniae* acquires essential Zn(II) cations and reveals the contribution of this element to bacterial physiology and virulence.

## Results

### *K. pneumoniae* AJ218 encodes two putative Zn(II)-associated ABC permeases

**Figure 1:**
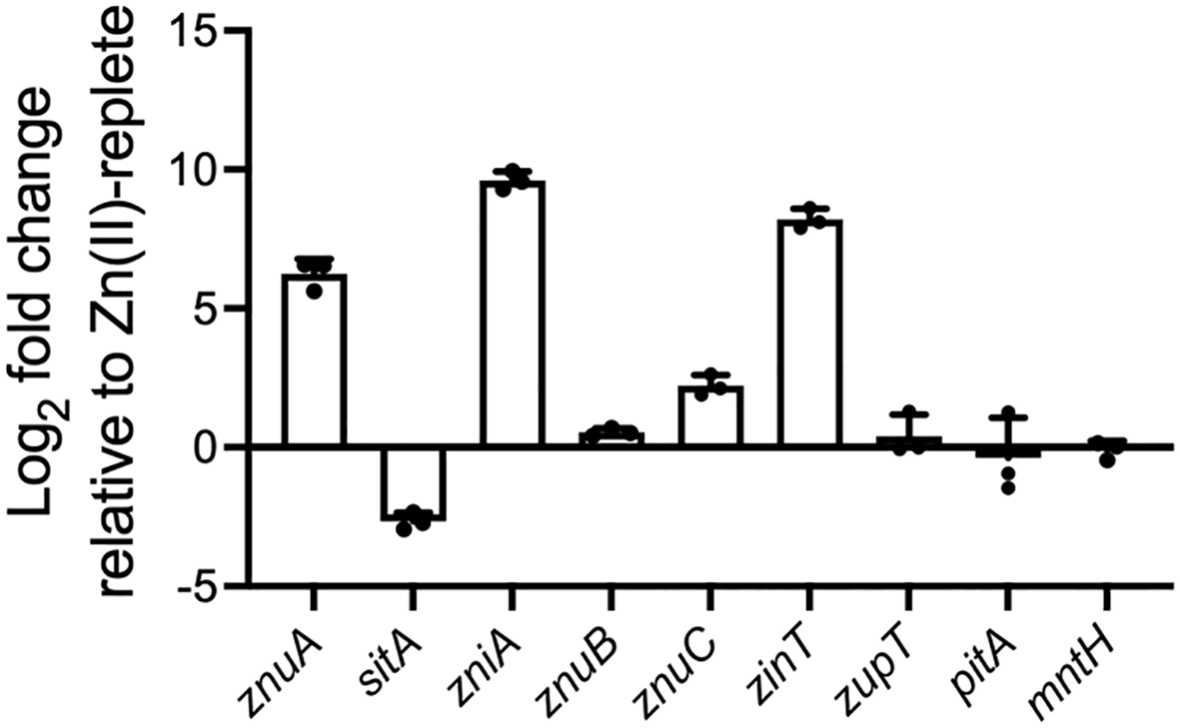
Relative expression of genes associated with putative metal uptake pathways during growth under Zn(II)-limitation. qRT-PCR of genes associated with putative metal uptake pathways in *K. pneumoniae* AJ218 pACYC184 grown in Zn(II)-limited or 10 µM ZnSO_4_ supplemented media. Data represent the mean (± SEM) log_2_-fold change in expression, in Zn(II)-limited media relative to -supplemented media, normalized to *rpoD*.

**Figure 2:**
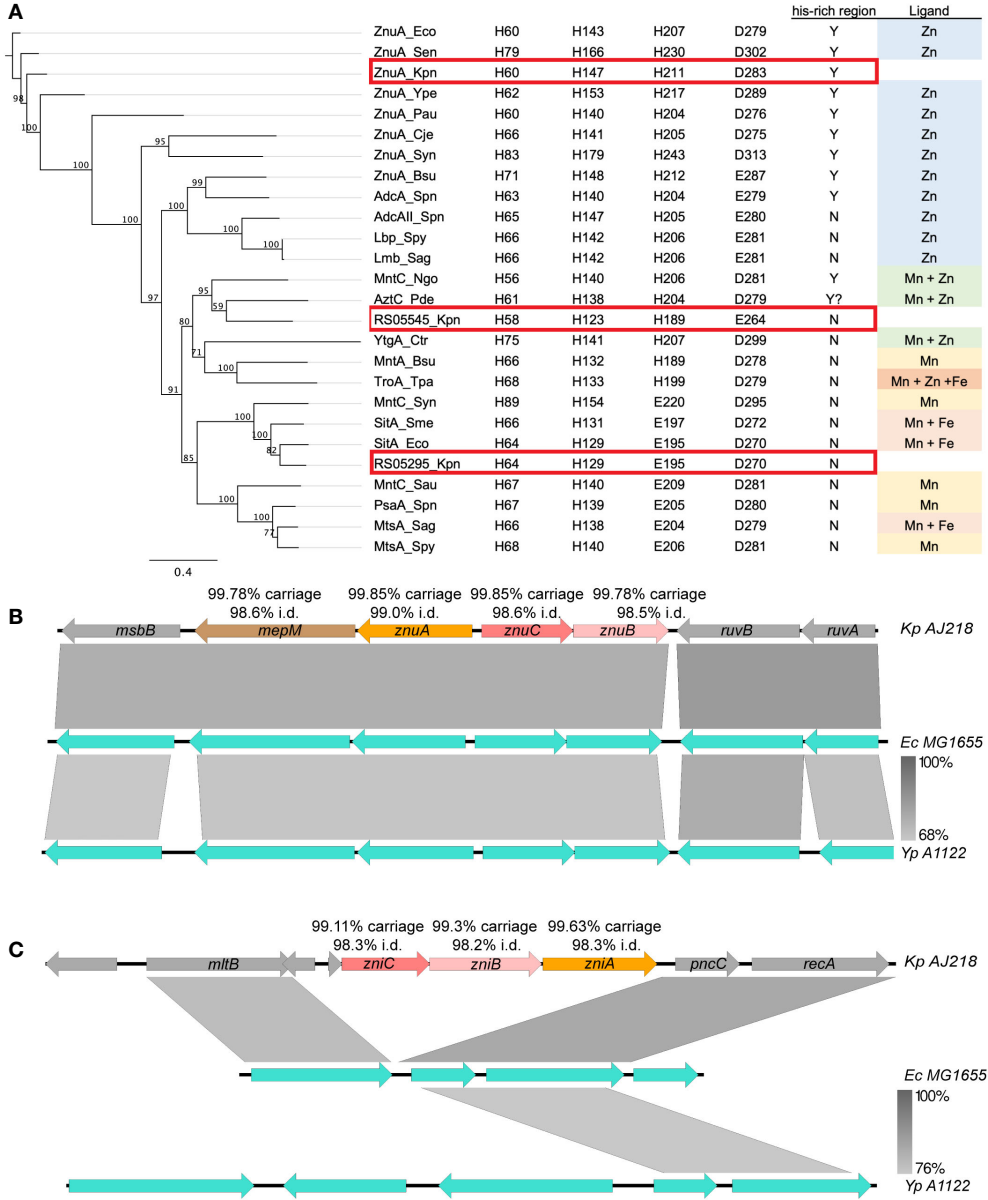
Genetic analysis of zinc import systems *znuCBA-mepM* and *zniCBA*. **(A)** Left: Phylogenetic analysis of ABC transporter substrate binding protein subunits from *K. pneumoniae* (Kpn, ZnuA, accession WP_004180437.1; RS05295, WP_004890185.1; and RS05545, WP_023286807.1), *Bacillus subtilis* (Bsu, WP_003229060.1, WP_003234733.1), *Campylobacter jejuni* (Cje, WP_014517164.1), *Chlamydia trachomatis* (Ctr, WP_009871416.1), *E. coli* (Eco, ABB58782.1, WP_001300644.1), *N. gonorrhoeae* (Ngo, WP_010951005.1), *P. aeruginosa* (Pau, NP_254185.1), *P. dentrificans* (Pde, WP_011747896.1), *Streptococcus agalactiae* (Sag, WP_000755138.1, WP_000759089.1), *S. aureus* (Sau, YP_499195.1), *S. enterica* serovar Typhimurium (Sen, WP_000939597), *Sinorhizobium meliloti* (Sme, WP_010970360.1), *S. pneumoniae* (Spn, WP_000724074.1, WP_001844050.1, WP_000733056.1), *Streptococcus pyogenes* (Spy, BAB69896.1, WP_004218965.1), *Synechocystis* sp. (Syn, BAA17919.1, BAA17110.1), *Treponema pallidum* (Tpa, AAC45725.1), *Y. pestis* (Ype, WP_002227944.1). Right: Conservation of key Mn/Zn binding residues and His-rich region, and ligand for SBP. The *K. pneumoniae* ZnuA, RS05545 and RS05295 are highlighted boxed in red. Local genomic comparisons (generated by EasyFig 2.0 (Sullivan et al., 2011)) of the **(B)** *znu* and **(C)** *zni* gene regions, in *K. pneumoniae* AJ218 (top, accession NZ_LR130541), *E. coli* MG1655 (middle, U00096) and *Y. pestis* A1122 (bottom, NC_017168) genomes. Genes are indicated by colored arrows with *K. pneumoniae* gene names inside and carriage rate and nucleotide identity statistics from *Klebsiella* spp. database screens given above (see **Supplementary Table 1** for more details). Where applicable, the levels of nucleotide homology between adjacent strains are indicated by the grey shading between loci, with scale bar indicated to the bottom right.

The *K. pneumoniae* AJ218 genome was investigated for putative Zn(II) import and homeostasis pathways using sequences of known systems from *E. coli* K12 MG1655, namely *znuCBA, zupT*, *zinT, pit*, and *mntH* (Elvin et al., 1986; Ferianc et al., 1998; Patzer and Hantke, 1998; Beard et al., 2000; Makui et al., 2000; Grass et al., 2002; Puškarova et al., 2002). Orthologs of all putative systems were identified in AJ218, and of these, substantial up-regulation of *znuA* and *zinT* was seen when *K. pneumoniae* AJ218 was grown in Zn(II)-limited media (≤80 nM ^66^Zn), relative to Zn(II)-supplemented media (10 µM ZnSO_4_, **Table 1**, **Figure 1**). The bioinformatic analyses also revealed the presence of two additional cluster A-I SBP orthologs, with intermediate amino acid sequence similarity (~55%) to *E. coli* MG1655 ZnuA, encoded by EW045_RS05295 and EW045_RS05545 (**Table 1**). To predict their putative functional roles, a phylogenetic analysis of the proteins was performed using functionally characterized cluster A-I SBPs, and sequences examined for motifs to determine potential metal ligand(s) (**Figure 2A**). *K. pneumoniae* AJ218 ZnuA clustered most closely with *E. coli* MG1655 ZnuA and other Gram-negative bacterial Zn(II)-specific SBPs (**Figure 2A**). In contrast, *K. pneumoniae* AJ218 EW045_RS05295 clustered most closely with SitA from *E. coli* APEC strain χ7122 (ABB58782; 90.79% similarity) and *Sinorhizobium meliloti* (WP_010970360.1; 89.70% similarity). Amino acid sequence analyses identified two histidine and two carboxylate residues as the putative metal-coordinating residues in EW045_RS05295, a combination frequently associated with manganese (Mn) and/or iron (Fe) recruitment (Eijkelkamp et al., 2015) (**Figure 2A**). Notably, an ortholog of this putative SBP has previously been characterized in *K. pneumoniae* NTUH-K2044, where it was designated SitA and shown to contribute to oxidative stress tolerance (Sun et al., 2014). In *K. pneumoniae* AJ218, expression of EW045_RS05295 decreased during growth in Zn(II)-limited media indicating a role distinct from Zn(II) import (**Table 1**, **Figure 1**). These data suggest that EW045_RS05295 likely contributes to Mn and/or Fe acquisition in *K. pneumoniae* AJ218. Accordingly, the SitA nomenclature assigned in *K. pneumoniae* NTUH-K2044 will be henceforth used.

**Table 1:** Putative *K. pneumoniae* AJ218 Zn(II) uptake determinants.

Name	Protein comparisons			Gene comparisons		
	*E. coli* ^1^	*K. pneumoniae* ^1^	Amino acid similarity (%)	*E. coli* ^1^	*K. pneumoniae* ^1^	Nucleotide identity (%)
ZnuA	AAC74927.2	WP_004180437.1	91.08	b1857	EW045_RS10020	73.0
SitA	AAC74927.2	WP_004890185.1	56.97	n/a	EW045_RS05295	–
ZniA	AAC74927.2	WP_023286807.1	54.68	n/a	EW045_RS05545	–
ZnuB	AAC74929.1	WP_004212745.1	98.8	b1859	EW045_RS10010	78.4
ZnuC	AAC74928.1	WP_002911449.1	98.85	b1858	EW045_RS10015	76.8
ZinT	AAC75039.1	WP_048269641.1	86.11	b1973	EW045_RS26395	59.0
ZupT	AAC76076.1	WP_038808242.1	96.89	b3040	EW045_RS03460	71.7
PitA	AAC76518.1	WP_002921200.1	97.6	b3493	EW045_RS01375	81.7
MntH	AAC75451.1	WP_040153932.1	97.82	b2392	EW045_RS07415	76.5

^1^Locus tags and protein accession numbers from *E. coli* MG1655 (U00096) and *K. pneumoniae* AJ128 (NZ_LR130541.1). n/a, not applicable (gene absent in genome).

Analyses of EW045_RS05545 revealed that it clustered most closely with the SBPs AztC from *Paracoccus denitrificans* (WP_011747896.1; 68.08% similarity), and MntC from *Neisseria gonorrhoeae* (WP_010951005.1; 66.24% similarity) (**Figure 2A**), which are involved in Zn(II)- and/or Mn(II)-recruitment (Tseng et al., 2001; Lim et al., 2008). However, the putative metal-coordinating residues were predicted to be a combination of three histidine and one carboxylate residues, an arrangement observed in *K. pneumoniae* AJ218 ZnuA and predominantly associated with Zn(II) recruitment. Expression of EW045_RS05545 was also significantly upregulated during growth in Zn(II)-limited media, and to a substantially greater extent than ZnuA (**Table 1**, **Figure 1**). This suggests that EW045_RS05545 is associated with *K. pneumoniae* AJ218 Zn(II) homeostasis and is more tightly regulated by cellular Zn(II) levels than the *znuCBA* system. Further, supporting this inference was co-location of the SBP gene within a putative operon that also encodes an ABC transporter (transmembrane domain (TMD), EW045_RS05540; nucleotide-binding domain (NBD), EW045_RS05535). This operon structure is frequently observed for ABC importers and their associated ligand-recruiting SBPs. Taken together, these data strongly suggest that *K. pneumoniae* AJ218 encodes a second Zn(II)-specific ABC permease that was designated as *zniCBA* and further investigated.

### ZnuCBA and ZniCBA are highly conserved in *Klebsiella* species

Conservation of the putative Zn(II)-associated ABC permeases was investigated using a database of 2,706 *Klebsiella* spp. genomes (Rocker et al., 2020). The prototypic Znu permease occurred as a four gene locus, with *znuA-mepM*, encoding the SBP and a murein DD-endopeptidase, transcribed divergently from *znuC-znuB*, the NBD and TMD, respectively. All genes were present in >99.7% of genomes analyzed, with nucleotide sequence conservation exceeding 98.5% pairwise identity (**Figure 2B**, **Supplementary Table 1**). This gene organization was also highly conserved in *Klebsiella* spp. genomes, with the locus carried in 98.4% of genomes, with 98.7% pairwise identity across the full length. The locus also shared 76.9% nucleotide identity to the orthologous *E. coli* MG1655 *mepM-znuCBA* genes and is highly conserved amongst the Enterobacteriaceae (**Figure 2B**, **Supplementary Figure 1A**).

The Zni permease was highly conserved within the *Klebsiella* genus, with the *zniCBA* genes present in ≥99.1% of genomes, with ≥98.2% nucleotide pairwise identity for each gene. The complete, unidirectional *zniCBA* locus was present in 97.0% of genomes with high conservation (98.2% pairwise identity) across the full locus (**Supplementary Table 1**). In contrast to the Znu permease, the *zniCBA* locus was only present in a small subset of Enterobacteriaceae, which included *Enterobacter cloacae* and *Kluyvera intermedia* (**Figure 2C**, **Supplementary Figure 1B**). The majority of representative Enterobacteriaceae spp. only contained the flanking genes, *mltB* and *pncC* (**Supplementary Figure 1B**). Given the differences in locus organization and the low nucleotide identity between the individual *znuCBA* and *zniCBA* genes (<50.1% pairwise identity) this suggests that the *zniCBA* operon was mobilized into this site, rather than a gene duplication of *znuCBA*.

### ZnuCBA and ZniCBA contribute to *K. pneumoniae* AJ218 Zn(II) acquisition

To define the contribution of the putative ZnuCBA and ZniCBA importers to *K. pneumoniae* AJ218 Zn(II) uptake, isogenic mutant strains lacking the genes encoding the SBPs in isolation (Δ*znuA*, *ΔzniA*) and combination (Δ*znuA ΔzniA*) were generated. The derivative strains were then complemented *in trans* using the low-copy plasmid pACYC184 in which *znuA* was expressed under its native promoter (pACYC184::*znuA*; hereafter, pZnuA) or *zniA* via the *tet* promoter (constitutive expression; pACYC184::*zniA*; hereafter, pZniA). The wild-type and derivative strains (containing the empty pACYC184 vector, or respective gene complements) were then assessed for their growth phenotypes and cellular accumulation of metal ions in Zn(II)-limited media, with or without 10 µM ZnSO_4_ supplementation.

**Figure 3:**
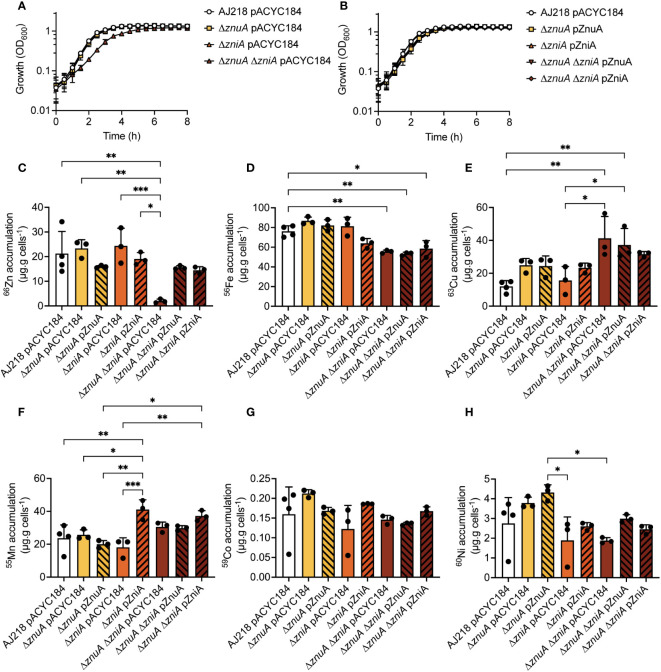
*K. pneumoniae* growth in Zn(II)-limited media and elemental content analyses. *K. pneumoniae* AJ218 pACYC184, mutant **(A)**, and complemented strains **(B)** growth phenotypes in Zn(II)-limited media. Data represent mean OD_600_ values (± SEM) from three independent experiments. Whole cell accumulation of ^66^Zn **(C)**, ^56^Fe **(D)**, ^63^Cu **(E)**, ^55^Mn **(F)**, ^59^Co **(G)**, and ^60^Ni **(H)** for the wild-type and derivative strains grown in Zn(II)-limited media. Data represent mean (± SEM) μg metal.g cells^-1^ (dry weight) from at least three independent experiments. Statistical significance of differences was determined by one-way ANOVA analysis with Bonferroni post-test; *, *P* < 0.05; **, *P* < 0.01; ***, *P* < 0.001.

The *K. pneumoniae* Δ*znuA* and Δ*zniA* and complemented strains had growth comparable to the wild-type strain in both Zn(II)-limited and Zn(II)-supplemented media (**Figures 3A, B**, **Supplementary Figures 2A, B**). Thus, these data show that loss of either SBP in isolation does not perturb growth of *K. pneumoniae* AJ218. In contrast, the *K. pneumoniae* Δ*znuA ΔzniA* strain showed a notable growth delay in Zn(II)-limited media, relative to the wild-type and the complemented strains, Δ*znuA* Δ*zniA* pZnuA and Δ*znuA* Δ*zniA* pZniA (**Figures 3A, B**). The growth rate of the Δ*znuA* Δ*zniA* strain was restored to essentially that of the wild-type strain upon supplementation with 10 µM ZnSO_4_ (**Supplementary Figures 2A, B**). These data indicate that during growth in Zn(II)-replete media, other pathways are sufficient to maintain the Zn(II) requirements of the cell. However, further studies would be required to determine the identity of these low affinity uptake systems.

The *K. pneumoniae* strains were then analyzed for whole cell Zn(II) accumulation during growth in Zn(II)-limited and supplemented conditions (**Figure 3C**, **Supplementary Figure 2C**). The *K. pneumoniae* Δ*znuA* and Δ*zniA* derivative and complemented strains accumulated ^66^Zn to levels comparable to the wild-type strain in both Zn(II)-limited and supplemented media (**Figure 3C**, **Supplementary Figure 2C**). Thus, loss of either SBP did not impair Zn(II) accumulation, consistent with the phenotypic growth analyses. However, loss of both SBPs in the *K. pneumoniae* Δ*znuA* Δ*zniA* strain significantly reduced ^66^Zn accumulation in Zn(II)-limited media relative to the wild-type, Δ*znuA*, and Δ*zniA* strains (*P <*0.0001, one-way ANOVA with Bonferroni post-test; **Figure 3C**). Complementation with either SBP or supplementation of the growth medium with 10 µM ZnSO_4_ restored Zn(II) accumulation to wild-type levels (**Figure 3C**, **Supplementary Figure 2C**). Collectively, these data show that ZnuCBA and ZniCBA contribute to *K. pneumoniae* AJ218 Zn(II) acquisition with either ABC permease necessary and sufficient to support wild-type growth and metal uptake.

### Dysregulation of Zn(II) uptake impacts metal homeostasis

Prior work has shown that disruption of Zn(II) homeostasis in *K. pneumoniae* via Zn(II) intoxication dysregulates the homeostasis of other *d*-block elements (Maunders et al., 2022). Building on that finding, the impact of Zn(II) limitation on the homeostasis of other metals was examined. Deletion of *znuA* or *zniA* in isolation did not significantly influence the homeostasis of other first-row *d*-block elements (namely, iron, ^56^Fe; copper, ^63^Cu; manganese, ^55^Mn; cobalt, ^59^Co; or nickel, ^60^Ni) during growth in Zn(II)-limited (**Figures 3D–H**) or Zn(II)-supplemented media (**Supplementary Figures 2D–H**). In Zn(II)-limited media, the Δ*znuA* Δ*zniA* strain showed no perturbations in ^55^Mn, ^59^Co, or ^60^Ni levels relative to wild type, but had significantly reduced ^56^Fe (*P =* 0.0046, one-way ANOVA with Bonferroni post-test; **Figure 3D**) and increased ^63^Cu (*P* = 0.0014, one-way ANOVA with Bonferroni post-test; **Figure 3E**) accumulation. Supplementation of the growth media with 10 µM ZnSO_4_ ameliorated the reduction in the Δ*znuA* Δ*zniA* ^56^Fe levels (**Supplementary Figure 2D**), whereas the ^63^Cu accumulation remained significantly elevated (*P* = <0.0001, one-way ANOVA with Bonferroni post-test; **Supplementary Figure 2E**). Interestingly, the baseline abundance of ^63^Cu was elevated in nearly all strains during growth in Zn(II)-limited conditions by comparison with Zn(II)-supplemented conditions (**Figure 3E**, **Supplementary Figure 2E**). The mechanisms behind these changes are not yet clear, but may be due to either dysregulation of ^63^Cu export, or increased ^63^Cu uptake into the cell. Although no Cu-specific uptake pathway has yet been identified in Enterobacteriaceae spp (Porcheron et al., 2013), the increase in ^63^Cu may represent adventitious uptake due to the use of alternate Zn(II) acquisition mechanisms by *K. pneumoniae*.

Unexpectedly, a significant increase in ^55^Mn accumulation was seen in the Δ*zniA* pZniA strain relative to the wild-type and Δ*zniA* pACYC184 strains during growth in Zn(II)-limited media (*P* = 0.0006 and *P* = 0.0056, respectively; one-way ANOVA with Bonferroni post-test; **Figure 3F**). Further, this increase in cellular ^55^Mn was also observed for the Δ*znuA* Δ*zniA* pZniA strain relative to the Δ*znuA* Δ*zniA* pACYC184 and Δ*znuA* Δ*zniA* pZnuA strains (*P* = 0.0015 and *P* = 0.0004, respectively; **Supplementary Figure 2F**) in Zn(II)-supplemented media. Taken together, these data show that constitutive expression of ZniA was associated with increased accumulation of Mn(II), but not other *d*-block elements. It therefore follows that ZniA may be able to interact with both Mn(II) and Zn(II) cations and facilitate their import. In contrast, the Δ*znuA* pZnuA strain showed a significant, albeit modest, reduction in ^55^Mn accumulation, relative to the wild type, during growth in Zn(II)-supplemented conditions (*P* = 0.0337, one-way ANOVA with Bonferroni post-test; **Supplementary Figure 2F**). However, this was not observed in the Δ*znuA* Δ*zniA* pZnuA complemented strain, so it is unclear whether this is due to the pZnuA construct. Collectively, these data show that Zn(II) depletion dysregulated Fe and Cu homeostasis, the underlying molecular basis of which warrants further investigation. Further, while ZnuA or ZniA is sufficient to overcome growth perturbation in Zn(II)-limited media, the association of ZniA with increased cellular Mn(II) suggests that there may be mechanistic differences between the two ABC permeases with respect to specificity of metal import. Given the unique identification of ZniA within a subset of the Enterobacteriaceae and its distinct interaction with metal ligands, its structural properties were investigated.

### Structural analyses of Zn(II)-bound ZniA

**Figure 4:**
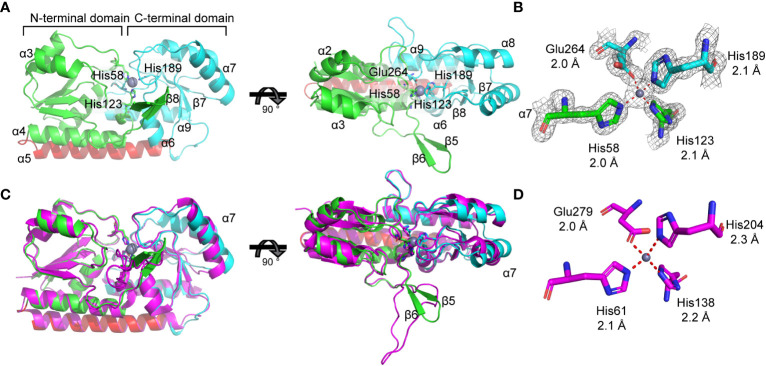
Biophysical analysis of ZniA. **(A)** Crystal structure of zinc-bound ZniA (PDB ID: 8SVC) showing the N-terminal lobe colored in green, the C-terminal lobe in cyan, the domain-linking helix in red and zinc as a grey sphere, from front (left) and top (right) views. **(B)** Tetrahedral coordination of zinc is mediated by residues His58, His123, His189, and Glu264, represented as sticks, with the density map overlayed and bond lengths indicated: 2.0 Å (His-58), 2.1 Å (His-123), 2.1 Å (His-189), and 2.0 Å (Glu-264). **(C)** Structural comparisons of *K. pneumoniae* ZniA with AztC from *P. denitrificans* (PDB ID 5W57; magenta), from front (left) and top (right) views. **(D)** AztC co-ordination residues, represented as sticks, with bond lengths indicated.

Recombinant, mature (residues 22-292) *K. pneumoniae* AJ218 ZniA was purified and crystallization was attempted using Mn(II) and Zn(II). Despite multiple attempts, Mn(II)-bound ZniA did not yield crystals of sufficient quality for X-ray diffraction. Crystals of the SBP in the Zn(II)-bound state were obtained and the structure determined at 1.59 Å resolution (**Figure 4A**, **Table 2**). A single ZniA molecule was present in the asymmetric unit with a fold characteristic of proteins belonging to the cluster A-I subgroup of ABC transporter-associated SBPs (Scheepers et al., 2016). ZniA was comprised by two globular domains (henceforth the N- and C-terminal domains), with each domain consisting of a central four-stranded parallel β-sheet surrounded by four α-helices. The N- and C-terminal domains were linked by an interdomain α-helix and the protein bisected by a cleft in which the metal binds (**Figure 4A**). The metal-binding site was located ~13 Å from the molecular surface of the protein and comprised by three Nϵ2 atoms, from His58 (β2α2 loop), His123 (β6α4 loop), and His189 (β7α6 loop), and one Oϵ2 atom, from Glu264 (β10α9 loop). These four residues bind a single Zn(II) ion via 4-coordinate geometry, with bond distances of 2.0 Å to 2.1 Å (**Figure 4B**). In the Zn(II)-bound state of ZniA, the metal-binding site was occluded from bulk solvent.

**Table 2:** Crystallographic data collection and refinement statistics.

Data collection^1^	ZniA
Space group	C 2 2 2_1_
Cell dimensions	
*a*, *b*, *c* (Å)	62.2, 141.3, 71.4
α, β, γ (°)	90.0, 90.0, 90.0
Wavelength (Å)	0.9201
Resolution (Å)	56.92 – 1.59 (1.647 – 1.59)
*R*_merge_	0.05 (0.55)
CC_1/2_	0.995 (0.547)
Average *I*/σ*(I)*	7.78 (1.37)
Completeness (%)	98.49 (95.34)
Multiplicity	2.0 (2.0)
Refinement	
Resolution (Å)	56.92 – 1.59 (1.647 – 1.59)
No. unique reflections	42008 (4014)
*R*_work_/*R*_free_ (%)	18.4/21.0
No. atoms	
Protein	1997
Ligand/ion	26
Water	341
B-factors (Å^2^)	
Protein	20.6
Ligand/ion	32.3
Water	31.4
R.m.s deviations	
Bond lengths (Å)	0.006
Bond angles (°)	1.12
Ramachandran statistics	
Favoured (%)	97.38
Allowed (%)	2.62

^1.^ Values in parentheses are for highest-resolution shell.

$R_{\text{merge}}=\sum_{\text{hkl}}\sum_{\text{j}}|I_{\text{hkl},\text{j}}-<I_{\text{hkl}}>|/(\sum_{\text{hkl}}\sum_{\text{j}}I_{\text{hkl},\text{j}})$

$R_{\text{work}}/R_{\text{free}}=\sum_{\text{hkl}}\left|F_{hkl}^{obs}-F_{hkl}^{calc}\right|/(\sum_{\text{hkl}}\;F_{hkl}^{obs})$ ; *R*_free_ was calculated using randomly chosen 10% fraction of data that was excluded from refinement.

The structure of Zn(II)-bound ZniA was next compared with the closest phylogenetic match, AztC from *P. denitrificans*, which has structural similarities to both Zn(II) and Mn(II) binding SBPs (**Figure 2A**). Structural superposition analyses revealed that the SBPs had similar global conformations, with a root mean square deviations of the Cα protein backbones (Cα-RMSD) of 1.3 Å (**Figure 4C**). Both ZniA and AtzC have binding sites that employ 3 histidine and 1 carboxylate residue for O_1_N_3_ tetracoordination of the metal ligand (**Figure 4D**). In AztC, this is associated with a substantial (>100-fold) preference for binding Zn(II) over Mn(II) (Handali et al., 2015), indicating a primary role for the SBP, and by extension ZniA, in Zn(II) transport. However, despite these similarities there remain structural differences between ZniA and AztC (**Figure 4C**). Structural superposition shows that helix α7 has different orientations between the two SBPs, while AztC also has a larger flexible loop compared to ZniA, which contains two β-sheets (β5,6) (**Figure 4C**). In many Zn(II)-specific SBPs, this flexible loop region frequently contains an extended, unstructured region enriched for histidine and other charged residues that promotes Zn(II) acquisition by the SBP. This region is relatively short in AztC, by comparison to other Zn(II)-specific SBPs (Handali et al., 2015), whereas this region is not enriched for histidine residues in ZniA.

Collectively, structural analyses of Zn(II)-bound ZniA show that it has an overall fold similar to other cluster A-I SBPs that interact with metal-importing ABC transporters. The coordinating residues are consistent with SBPs that show a preference for Zn(II) relative to Mn(II), but further studies will be required to comprehensively define the biophysical and metal interaction properties of this protein relative to *P. denitrificans* AztC, and other cluster A-I SBPs.

### Oxidative stress tolerance is impaired by Zn(II) limitation

**Figure 5:**
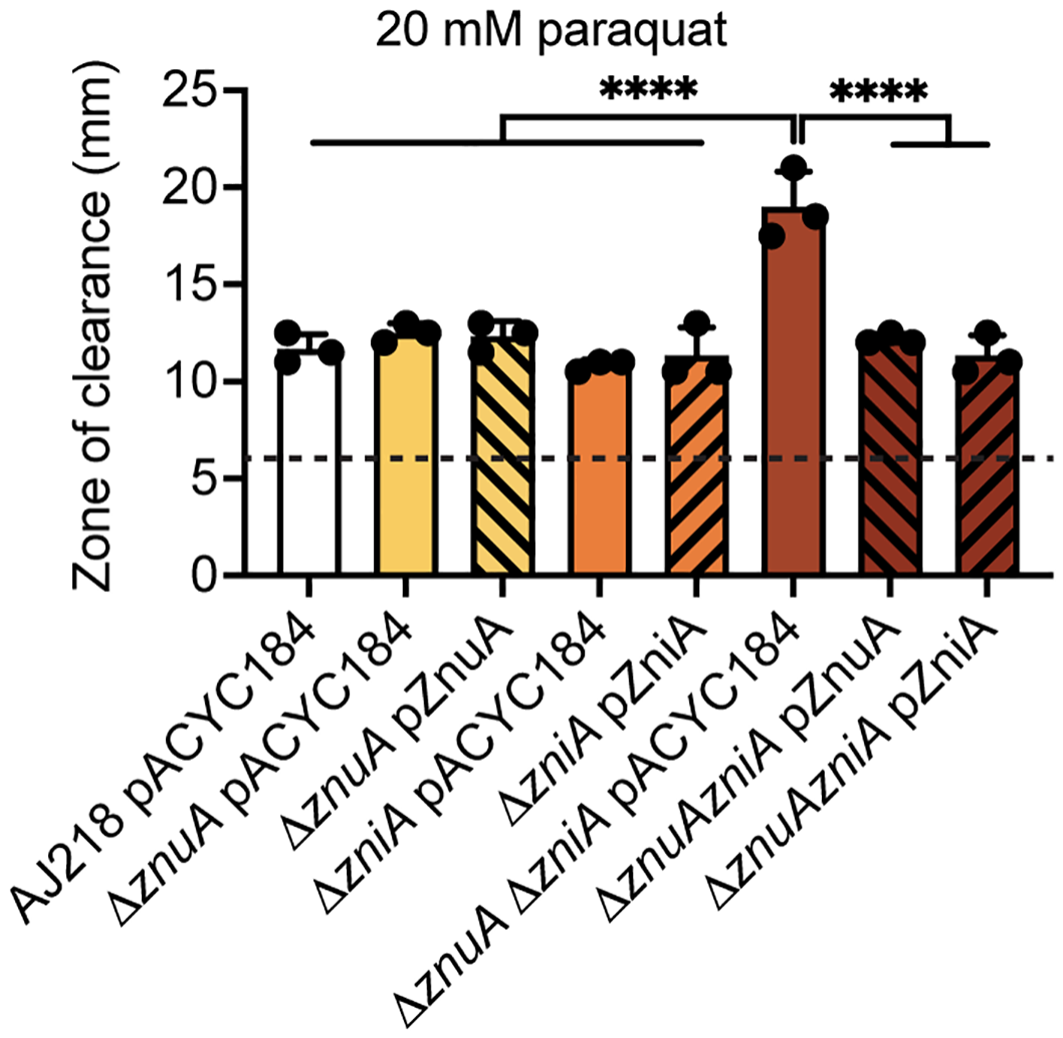
Oxidative stress of *K. pneumoniae* under Zn(II)-limitation. Zone of clearance measurements of the wild-type and derivative strains grown on Zn(II)-limited media in the presence of 20 mM paraquat impregnated discs. Data represent mean (± SD) clearance from three independent experiments, with limit of detection (disc size) shown by the dotted line. Statistical significance of differences between strains compared to the Δ*znuA* Δ*zniA* pACYC184 strain were determined by one-way ANOVA analysis with Tukey post-test. ****, *P* < 0.0001.

Impairment of Zn(II) acquisition has been associated with perturbed oxidative stress tolerance in diverse bacterial species including *N. gonorrhoeae* and *E. coli* (Seib et al., 2006; Seo Sang et al., 2015). In *E. coli*, *znuCBA* expression, has been implicated in aiding bacterial oxidative stress tolerance, via induction by oxidative stress regulators OxyR, SoxR, and SoxS systems (Nunoshiba et al., 1992; Zheng et al., 1998; Seo Sang et al., 2015) in the presence of viologen N,N’-dimethyl-4,4’-bipyridinium dichloride (paraquat). Paraquat is a redox compound that futilely cycles with low-potential electron donors and molecular oxygen in the cell to generate cytoplasmic superoxide, which is typically detoxified by superoxide dismutase metalloenzymes. Three superoxide dismutases are encoded in *K. pneumoniae*, of which SodC is Cu/Zn-dependent (Najmuldeen et al., 2019). Using disc diffusion assays, the Δ*znuA* Δ*zniA* strain was observed to be significantly more susceptible to paraquat exposure than the wild-type, single mutant, and complement strains (P<0.0001, one-way ANOVA with Turkey post-test, **Figure 5**). Whether this was due to a direct impairment of SodC activity, perturbation of cellular membrane and/or cell wall integrity, or dysregulation of Fe and/or Cu homeostasis and related pathways remains to be fully elucidated.

### Zn(II) limitation alters *K. pneumoniae* cell morphology

**Figure 6:**
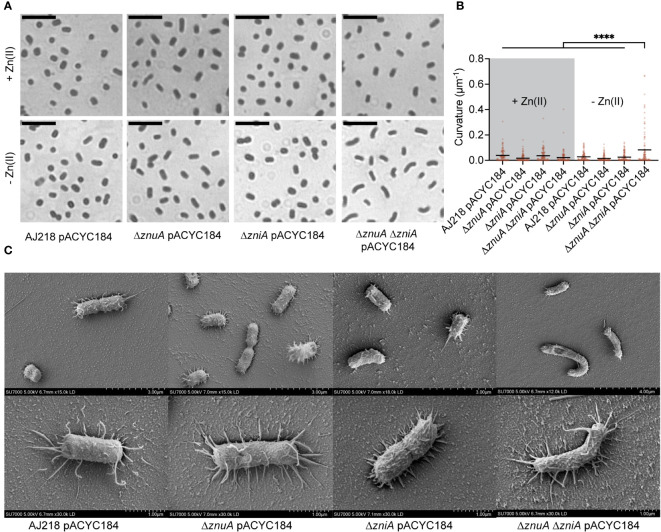
Morphological variation in *K. pneumoniae* under Zn(II)-limitation. **(A)** Representative images of *K. pneumoniae* AJ218 wild-type and derivative strains grown in Zn(II)-limited media in the presence or absence of 10 µM ZnSO_4_, stained with crystal violet. Scale bar (5 µM) is shown. **(B)** Curvature analyses of the strains using Fiji plugin MicrobeJ. Statistical significance of differences was determined by one-way ANOVA analysis with Tukey post-test. ****, *P* < 0.0001. **(C)** Representative images of the wild-type and derivative strains grown in Zn(II)-limited media visualized at 5 kV by field emission scanning electron microscopy. Scale bars are shown.

The impact of Zn(II) limitation, and the pleiotropic effects on metal homeostasis, on the physiology of *K. pneumoniae* AJ218 were further investigated. Microscopic examination of the Δ*znuA* Δ*zniA* strain grown in Zn(II)-limited media showed greater curvature than the wild type and single mutant strains, while this effect was ablated by Zn(II) supplementation (**Figures 6A, B**, **Supplementary Figures 3A, B**). Scanning electron microscopy analyses showed that curvature of Zn(II)-limited Δ*znuA* Δ*zniA* cells was due to morphological variation of individuals cells, rather than due to incomplete separation of two attached cells (**Figure 6C**, **Supplementary Figure 3C**). Thus, these data show that Zn(II) limitation impairs *K. pneumoniae* AJ218 cell wall and/or cell division processes.

**Figure 7:**
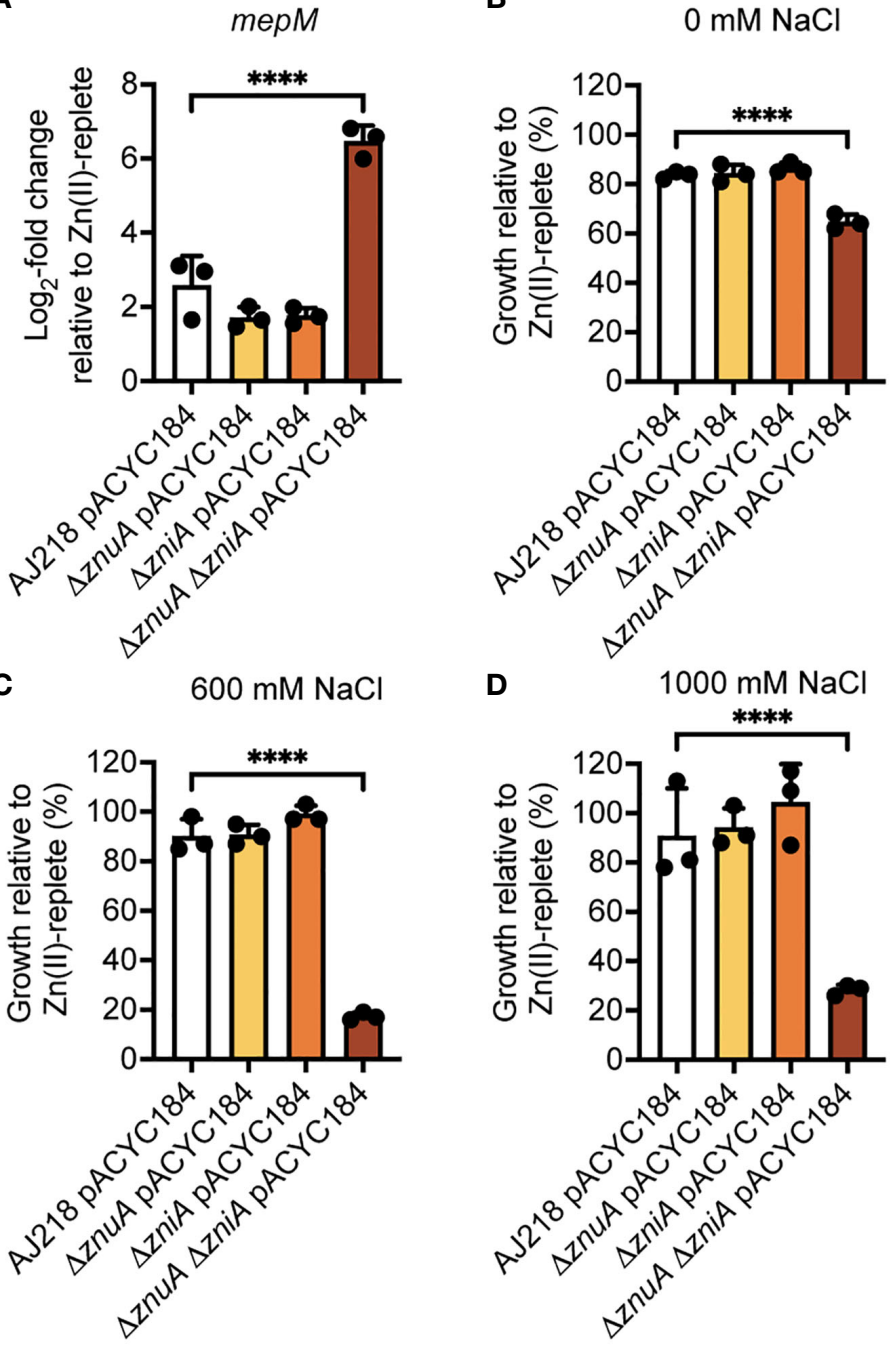
Phenotypic analysis of *K. pneumoniae* under Zn(II)-limitation. **(A)** qRT-PCR analysis of *mepM* in wild-type and derivative strains of *K. pneumoniae* AJ218 carrying pACYC184, grown in the presence or absence of 10 µM ZnSO_4_ from three independent experiments. Data represent mean (± SEM) log_2_-fold change in gene expression in Zn(II)-limited compared to -supplemented conditions, normalized to *rpoD*. **(B–D)** Salt stress assays of wild-type and derivative strains grown in the presence or absence of 10 µM ZnSO_4_. Data represent mean (± SEM) growth at 24 h in **(B)** 0 mM, **(C)** 600 mM, or **(D)** 1000 mM NaCl relative to Zn(II)-supplemented growth in three independent experiments. Statistical significance of differences between strains compared to the Δ*znuA* Δ*zniA* pACYC184 strain were determined by one-way ANOVA analysis with Dunnett post-test. ****, *P* < 0.0001.

Although the molecular basis for the alteration in cell morphology remains to be elucidated, one factor may be the murein DD-endopeptidase MepM, which is encoded downstream of *znuA* in Enterobacteriaceae spp. (**Figure 2B**, **Supplementary Figure 1A**). In *E. coli*, MepM has been shown to associate with the penicillin binding proteins Pbp1a and Pbp1b and contribute to peptidoglycan turnover and renewal of the cell wall (Park et al., 2020). Impairment of MepM activity, and that of the other murein DD-endopeptidases in *E. coli*, has been shown to perturb cell morphology and potentially abrogate cell viability. The altered cell morphology of the Δ*znuA* Δ*zniA* strain during Zn(II) limitation suggested that MepM endopeptidase activity may also be disrupted under these conditions. Accordingly, expression of *mepM* during growth in Zn(II)-limited conditions was assessed for the *K. pneumoniae* strains. This revealed upregulation of *mepM* in all strains, relative to Zn(II)-replete conditions, and to a significantly greater extent in the Δ*znuA* Δ*zniA* strain by comparison to the wild type (*P* < 0.0001, one-way ANOVA with Dunnett post-test; **Figure 7A**). In *E. coli*, MepM has been reported to be a Zn(II)-dependent metalloprotein with the metal cofactor required for function (Singh et al., 2012; Park et al., 2020). It therefore follows that the reduced cellular Zn(II) in the Δ*znuA* Δ*zniA* strain may impair endopeptidase activity despite the upregulation of *mepM* expression. This inference was investigated using a salt stress resistance assay, which has previously been used to report on MepM activity in *E. coli* (Park et al., 2020). Accordingly, the tolerance of the *K. pneumoniae* strains to salt exposure was investigated. All strains showed a modest decrease in absolute growth in Zn(II)-limited media relative to Zn(II)-supplemented media, with the Δ*znuA* Δ*zniA* strain showing significantly greater growth perturbation relative to the wild type (*P* < 0.0001, one-way ANOVA with Dunnett post-test; **Figure 7B**). Increasing levels of salt stress had no impact on the relative survival of the wild type or single mutant strains (**Figures 7C, D**). In contrast, the Δ*znuA* Δ*zniA* strain showed potent inhibition in relative growth and was significantly reduced relative to the wild type strain (**Figures 7C, D**). Taken together, these data show although *mepM* expression was upregulated by Zn(II) limitation, in the Δ*znuA* Δ*zniA* strain, the reduction in tolerance to salt stress phenocopies the impact reported in an *E. coli* Δ*mepM* strain (Park et al., 2020). These findings suggest that in the Δ*znuA* Δ*zniA* strain, MepM activity may be perturbed, most likely due to the lack of Zn(II) for the endopeptidase, and this may have contributed to aberrant cell morphology. However further investigations will be required to determine the veracity of this inference.

### Zn(II) acquisition contributes to *K. pneumoniae* virulence

**Figure 8:**
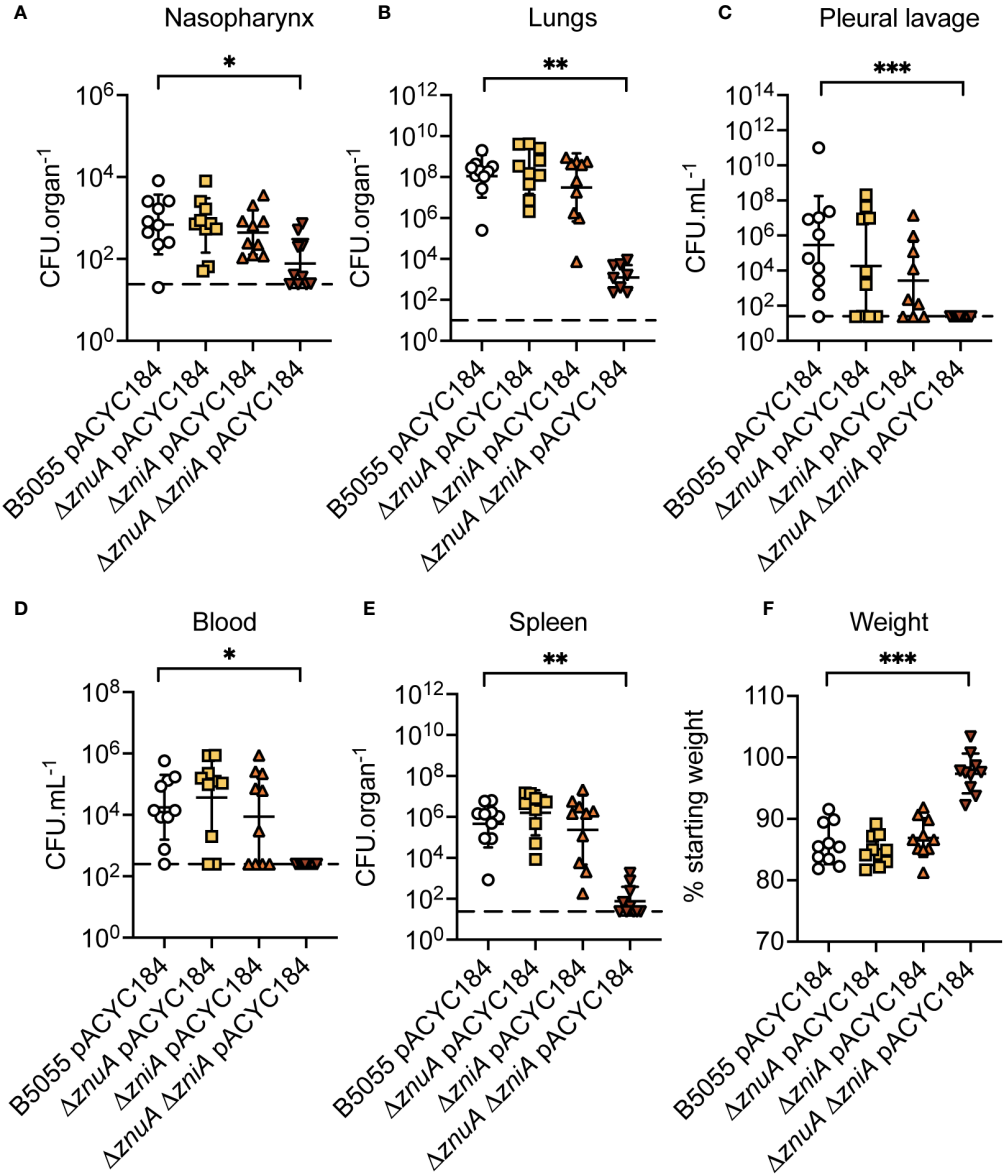
*K. pneumoniae* B5055 virulence in a murine model of lung infection. *K. pneumoniae* B5055 wild-type and derivative strain burden at 36 h post infection in murine niches: **(A)** nasopharynx, **(B)** lung, **(C)** pleural cavity, **(D)** blood, and **(E)** spleen. Each data point represents colony forming units (CFU) enumerated from one mouse (n=10) with geometric mean (± SD) presented as CFU.organ^-1^ **(A, B, E)** or CFU.mL^-1^ **(C, D)**. Dotted lines represent the limit of detection. **(F)** represents geometric mean (± SD) of weight loss (%), relative to starting weight. Statistical significance of differences was determined by non-parametric Kruskal-Wallis testing, corrected for multiple comparisons with Dunn’s test. ns, not significant; *, *P* < 0.05; **, *P* < 0.01; ***, *P* < 0.001.

The essentiality of Zn(II) for bacterial virulence is exploited by the innate immune response of vertebrates by a variety of antimicrobial strategies (Corbin et al., 2008; Eijkelkamp et al., 2019; Murdoch and Skaar, 2022). Accordingly, the contribution of the ZnuCBA and ZniCBA permeases to *K. pneumoniae* virulence in a murine lung infection model was investigated. Due to the limited murine virulence associated with *K. pneumoniae* AJ218, this was addressed by generating *ΔznuA*, *ΔzniA*, and *ΔznuA ΔzniA* derivative strains in the hypervirulent *K. pneumoniae* B5055 strain (Clements et al., 2007). The B5055 derivative strains showed comparable *in vitro* phenotypes to their respective AJ218 strains, with the B5055 *ΔznuA ΔzniA* strain similarly demonstrating impaired growth and altered cell morphology relative to the parental strain in Zn(II)-limited media (**Supplementary Figure S4**). The B5055 strains were then used to determine the contribution of the Zn(II) uptake pathways to *K. pneumoniae* virulence via intranasal infection of BALB/C mice. At 36 h post infection, mice were assessed for signs of clinical infection (e.g. ruffled appearance and weight loss), humanely sacrificed, and tissues harvested to determine the bacterial burden (**Figure 8**). In murine niches infected by *K. pneumoniae* B5055, loss of ZnuA or ZniA alone did not significantly alter bacterial burden (**Figures 8A–E**). In contrast, the *ΔznuA ΔzniA* strain had significantly reduced burden relative to the wild type in all niches. The data suggest that disruption of *K. pneumoniae* Zn(II) acquisition impaired nasopharyngeal colonization and/or compromised bacterial resistance to clearance from this niche (**Figure 8A**). Bacterial burden was also significantly reduced within the lungs (**Figure 8B**), with dissemination into deeper tissues, the pleural cavity, blood, and spleen (**Figures 8C–E**), dramatically compromised. The impaired virulence of the B5055 *ΔznuA ΔzniA* strain was also consistent with the lack of weight loss in this cohort (<3%) relative to the other infection groups (13-16% reduced weight; **Figure 8F**). Although complemented strains were not used in this study, collectively, these data show that Zn(II) acquisition is crucial for *K. pneumoniae* virulence in a murine model of infection, and can be mediated by strains with either the Znu (Δ *zniA*) or Zni (Δ *znuA*) permease.

## Discussion

Zn(II) is an essential element for the structure and function of up to 6% of the bacterial proteome (Andreini et al., 2006) and the second most abundant *d*-block metal in *K. pneumoniae* (Maunders et al., 2022). This work has identified that *K pneumoniae* AJ218 encodes two high affinity ABC permeases associated with Zn(II) homeostasis, ZnuCBA and ZniCBA. Although the ZnuCBA system is highly conserved in various prokaryotes, the ZniCBA system is a novel second Zn(II)-associated ABC permease of unknown origin, that is highly conserved in a subset of Enterobacteriaceae. ZniA structural analyses revealed the protein has a canonical cluster A-I SBP fold and contains a single metal binding site that tetrahedrally coordinates a Zn(II) ion via three histidine and one glutamate residues.

Many bacterial species employ multiple pathways to acquire Zn(II) such as *P. denitrificans* (Neupane et al., 2019), *Listeria monocytogenes* (Corbett et al., 2012), non-typeable *Haemophilus influenzae* (Rosadini et al., 2011), and *Vibrio cholerae* (Sheng et al., 2015). Recent studies have also revealed that some bacterial species, including *Staphylococcus aureus* (Grim et al., 2017)*, Yersinia pestis* (Bobrov et al., 2014), and *Pseudomonas aeruginosa* (Lhospice et al., 2017), can employ metallophores to recruit Zn(II) in a manner similar to siderophore-mediated Fe scavenging. For bacterial pathogens, this can be attributed to the essentiality of Zn(II) acquisition during infection and the innate immune response that targets this process (Murdoch and Skaar, 2022). Loss of Zn(II)-specific ABC permeases can exert a profound impact on the virulence of diverse pathogens including *Streptococcus pneumoniae* (Plumptre et al., 2014), *S. enterica* (Ammendola et al., 2007), *Acinetobacter baumannii* (Hesse et al., 2019), and *Moraxella catarrhalis* (Murphy et al., 2013). However, loss of Zn(II)-specific ABC permeases can be tolerated in pathogens that also encode alternative Zn(II)-acquiring systems, such as *Y. pestis*, which can employ yersiniabactin (Bobrov et al., 2014), and *S. aureus*, which can use the staphylopine-Cnt system (Grim et al., 2017). This work showed that the *in vivo* fitness of the *K. pneumoniae* B5055 Δ*znuA* Δ*zniA* strain was severely perturbed at 36 h post-infection, but that either Zn(II)-specific ABC permease was sufficient for full virulence. Despite the apparent redundancy of the two systems, the transcriptional analyses showed that *zniA* was induced to substantially greater extent than *znuA*, during growth in Zn(II)-limited media. This suggests that in *K. pneumoniae* AJ218 *zniA* and *znuA* are regulated differently by cellular Zn(II) abundance. Differential regulation of Zn(II) import components has previously been shown to be important for metal acquisition at different stages of host infection in *S. pneumoniae* (Plumptre et al., 2014). Alternately, the transporters may serve niche-specific roles, as exemplified by *L. monocytogenes*, where Zn(II) acquisition by ZurAM, but not ZinABC, supports intracellular growth (Corbett et al., 2012). While the ZnuCBA and ZniCBA permeases appeared functionally redundant in acute lung infection models, further investigation will reveal whether these systems have distinct contributions to invasion of other Zn(II)-restricted host niches, such as the gut or urinary tract, or enhances the competitive advantage of pathogen in polymicrobial environments.

One difference between the two ABC permeases is the potential capacity for ZniCBA to transport Mn(II) cations in addition to its primary role in Zn(II) homeostasis. This inference is supported by the phylogenetic analysis of ZniA wherein the protein grouped most closely with SBPs that have reported interactions with both cations, such as *N. gonorrhoeae* MntC (Lim et al., 2008). Further, structural analysis of ZniA showed that the protein lacks the histidine-enriched loop region common to Zn(II)-specific SBPs, including the highly similar ortholog AztC (Handali et al., 2015), but absent from Mn(II) and Fe(II)-specific SBPs. However, the role of Mn(II) in *K. pneumoniae* and the essentiality of this element for viability and virulence remain to be fully defined. Irrespective, it is unlikely that ZniCBA serves a major role in Mn(II) acquisition as its expression is regulated by Zn(II), and *K. pneumoniae* also encodes a putative Mn(II)-specific SBP, SitA, orthologs of which have been established to contribute to Mn(II) uptake in other Enterobacteriaceae spp. Nevertheless, expression of *zniA* during growth in Zn(II)-limited conditions may contribute to the efficacy of Mn(II) acquisition in these conditions. Future investigations, including defining the metal interaction properties of *K. pneumoniae* ZnuA, ZniA, and SitA, will aid in addressing these open questions.

Disruption of Zn(II) homeostasis also perturbed Fe and Cu homeostasis. The decrease in cellular Fe in the Δ*znuA* Δ*zniA* strain was readily reversed by 10 µM ZnSO_4_ supplementation, indicating that this was, directly or indirectly, due to the reduction in cellular Zn(II). Although the molecular basis for this impairment remains to be determined, one possible mechanism could be via the Fe-responsive ferric uptake regulator Fur that, in *E. coli*, requires Zn(II) as a structural cofactor (Althaus et al., 1999). The reduction in cellular Zn(II) may prevent cofactor acquisition by the protein resulting in poor or disrupted folding of Fur thereby leading to inappropriate derepression of the Fur regulon. In contrast, Cu accumulation increased during growth in Zn(II)-restricted conditions, most notably in the *K. pneumoniae* Δ*znuA* Δ*zniA* strain. However, while this was reduced in all strains upon 10 µM ZnSO_4_ supplementation, it remained elevated in the Δ*znuA* Δ*zniA* strain relative to the wild type. The molecular basis for Cu uptake in the Enterobacteriaceae remains to be fully defined, as no specific transporter has yet been identified. Current models suggest that Cu uptake occurs via other elemental or nutrient uptake pathways that are permissive for interaction with the metal. Recent studies of *E. coli* UTI89 and *S. aureus* have suggested that Cu uptake may be mediated by metallophores, such as yersiniabactin (Koh et al., 2017) or staphylopine (Hossain et al., 2023). While the number and type of metallophores produced by *K. pneumoniae* AJ218 remain to be determined, *K. pneumoniae* strains can contain up to four known siderophores, including yersiniabactin (Wyres et al., 2020). Although yersiniabactin is primarily associated with Fe transport, it has also been associated with Zn(II) uptake in *Y. pestis* (Bobrov et al., 2014) and *E. coli* (Behnsen et al., 2021). Accordingly, the increased Cu accumulation in the Δ*znuA* Δ*zniA* strain may reflect the use of similar pathways primarily employed to mitigate Zn(II) limitation. Taken together, these data highlight that Zn(II) limitation in *K. pneumoniae* AJ218 impacts the homeostasis of other essential *d-*block elements in a manner distinct from other bacterial pathogens (Plumptre et al., 2014; Pederick et al., 2015; Sheng et al., 2015) and further characterization of *K. pneumoniae* metallophores and their binding properties may be needed to determine their exact contribution to metal homeostasis. However, it is important to note that the experiments conducted herein focused on *K. pneumoniae* during exponential phase growth where Zn(II) dependency is anticipated to be greatest. The impact of Zn(II) limitation on *K. pneumoniae* in other growth phases, such as stationary phase, remain to be defined and could reveal further complexity.

Disruption of Zn(II) uptake can also compromise bacterial fitness due to the impact on Zn(II)-dependent metalloproteins. One manifestation is aberrant cell morphology, which has been reported for pathogens including *S. pneumoniae*, wherein dysregulation of Zn(II) and Mn(II) levels has been implicated in perturbing the metallation status of cell division regulatory machinery (Martin et al., 2017), and *A. baumannii*, due to altered activity of Zn(II)-dependent endopeptidases (Lonergan et al., 2019; Kim et al., 2021). In some bacterial species, Zn(II) limitation induces the expression of alternative endopeptidases, such as ShyB in *V. cholerae*. ShyB activity was shown to prevent aberrant *V. cholerae* cell morphology upon exposure to a Zn(II)-chelating agent, implying it compensates for the loss of ShyC activity, one of the two primary endopeptidases (Murphy et al., 2019). In *E. coli*, MepM is reported to be a Zn(II)-dependent endopeptidase, although some reports indicate calcium and magnesium can also restore enzymatic activity (Singh et al., 2012; Park et al., 2020). In *K. pneumoniae*, *mepM* expression is upregulated during Zn(II)-limitation, which supports the inference that it may serve as an alternative endopeptidase, such as ShyB, during growth in nutritionally deficient conditions (Murphy et al., 2019). However, during Zn(II) limitation the Δ*znuA* Δ*zniA* strain has increased sensitivity to salt stress, phenocopying the impact of a *mepM* deletion in *E. coli* (Park et al., 2020). Given the aberrant cellular morphology and reduced salt stress, it is plausible to speculate that activity of the endopeptidase is perturbed in these conditions; however there remains a lack of direct data on *K. pneumoniae* MepM activity. Further insight into the underlying molecular basis of altered cell morphology, bacterial Zn(II) homeostasis, and MepM endopeptidase activity warrants investigation.

Collectively, this study reports new insights into *K. pneumoniae* Zn(II) homeostasis and the ABC permeases that contribute to this process. Notably, this work revealed the contribution of the novel ZniCBA permease, which is highly conserved in *Klebisella* spp. and a subset of the Enterobacteriaceae, and provided the first structural analysis of the metal-recruiting SBP component. This work established that the two ABC permeases appeared to be functionally redundant, while loss of both systems exerted pleiotropic impacts on *d*-block metal homeostasis, cell morphology, and tolerance to chemical stresses. Given the demonstrable importance of Zn(II) acquisition for *in vivo* virulence of *K. pneumoniae*, these findings provide a robust foundation for future investigations of Zn(II)-dependent processes in the pathogen to identify potential targets for novel anti-infective development strategies.

## Materials and methods

### Bioinformatic analyses

Putative *K. pneumoniae* Zn(II) uptake mechanism genes were identified by TBLASTN analysis of the AJ218 genome (accession NZ_LR130541.1) with *E. coli* K12 MG1655 candidates (**Table 1**), using the BLAST plugin in Geneious Prime (Geneious version 2022.1, Biomatters) with default settings. For SBP comparison and phylogeny, translated full-length protein sequences, as reported in the text, were used for variation analysis by MUSCLE alignment (v 3.8.425) (Edgar, 2004) in Geneious Prime with default settings, with BLOSUM 45 and threshold of 0 parameters used for reporting similarity scores. Key binding residues and His-rich loop were identified from alignments, relative to residues previously determined (Eijkelkamp et al., 2015). Phylogenetic trees were generated using the Geneious Tree Builder with Jukes-Cantor genetic distance model and Neighbor-Joining with no outgroup. To assess conservation, a database of 2,706 publicly available *Klebsiella* genomes (Rocker et al., 2020) was screened for putative Zn(II) uptake genes using the BLASTN screening tool, Screen Assembly (v1.2.7) (Davies et al., 2019), applying cut-offs of 80% identity and 80% reference length. Gene absences were further validated by screening 3 × 240 bp segments for each target gene (*znuA* and *zniA*) (**Supplementary Table 1**). Local genome comparisons were carried out in EasyFig (Sullivan et al., 2011), using local BLASTN analysis of genomes as indicated in the text.

### Bacterial strains, chemicals, and media

Bacterial strains and plasmids used in this study are listed in **Table 3**. *K. pneumoniae* strains were routinely cultured via overnight growth in M9 media (Cold Spring Harbor Laboratory, 2010) (0.49 μM ^66^Zn), followed by growth in Zn(II)-limited or -supplemented M9 media for assays. Zn(II)-limited media was prepared by overnight agitation of M9 media with 5% (w/v) Chelex-100 resin (Bio-Rad) at pH 7.0. The Chelex-100 treated medium was filter sterilized and supplemented with 1 mM MgSO_4_, 0.027 mM CaCl_2_, 0.077 µM MnSO_4_, 0.297 µM FeSO_4_, 0.182 µM NiCl_2_, and 0.610 µM CuSO_4_. Zn(II)-limited media had a ^66^Zn concentration of 0.075 µM. Zn(II)-supplemented media was prepared by addition of 10 µM ZnSO_4_ to Zn(II)-limited media. For growth experiments, *K. pneumoniae* strains were harvested from overnight cultures by centrifugation at 18,000 × *g* for 5 min at room temperature and then resuspended to an optical density at 600 nm (OD_600_) of 0.05 in Zn(II)-limited or Zn(II)-supplemented media. Strains were then grown with aeration at 300 rpm in biological triplicate at 37°C in a FLUOStar Omega spectrophotometer (BMG Labtech). Zn(II)-limited agar plates were prepared using 2 × concentrated Zn(II)-limited media mixed with an equivalent volume of 3% (w/v) bacteriological agar (Oxoid). Culture media was supplemented, where appropriate, with the antibiotics kanamycin (Km, 50 μg.mL^-1^) and/or chloramphenicol (Chl, 20 μg.mL^-1^ for *E. coli*, 80 μg.mL^-1^ for *K. pneumoniae*).

**Table 3:** Strains and plasmids used in this study.

Strain	Genotype or description	Reference
NEB5-α	High efficiency competent *E. coli*, *fhuA2 (argF-lacZ)*U169 *phoA glnV44* 80 *(lacZ)*M15 *gyrA*96 *recA1 relA1 endA1 thi-1 hsdR17*	New England Biolabs
BL21(DE3)	*fhuA2 [lon] ompT gal (λ DE3) [dcm] ΔhsdS λ DE3 = λ sBamHIo ΔEcoRI-B int::(lacI::PlacUV5::T7 gene1) i21 Δnin5*	New England Biolabs
AJ218	Wild-type *Klebsiella pneumoniae* clinical isolate, serotype K54, Ap^R^	(Wilksch et al., 2011)
AJ218 Δ*znuA*	AJ218 deletion mutant *znuA*; ampicillin resistant (Ap^R^)	This study
AJ218 Δ*zniA*	AJ218 deletion mutant *zniA::Km*; Ap^R^ Km^R^	This study
AJ218 Δ*znuA* Δ*zniA*	AJ218 deletion mutant *znuA; zniA::Km*; Ap^R^ Km^R^	This study
B5055	Wild-type *Klebsiella pneumoniae* clinical isolate*;* mouse lethal, serotype K2:O1 Ap^R^	(Clements et al., 2007)
B5055 Δ*znuA*	B5055 deletion mutant *znuA*; Ap^R^	This study
B5055 Δ*zniA*	B5055 deletion mutant *zniA::Km*; Ap^R^ Km^R^	This study
B5055 Δ*znuA* Δ*zniA*	B5055 deletion mutant *znuA; zniA::Km*; Ap^R^ Km^R^	This study
Plasmid	Feature or description	Reference
pACYC184	Low-copy-number cloning vector, p15A ori; Tetracycline^R^ Chl^R^	New England Biolabs
pKD4	FRT-flanked Km^R^ cassette; Ap^R^ Km^R^	(Datsenko and Wanner, 2000)
pACBSR	Ara promoter control, I-SceI and l Red recombinase; Chl^R^	(Herring et al., 2003)
pFLP-BSR	pACBSR derivative, containing Flp enzyme from pCP20; Chl^R^	(Cherepanov and Wackernagel, 1995), gift from R.A. Strugnell
pZnuA	pACYC184::ZnuA – *K. pneumoniae* AJ218 *znuA* cloned into Chl^R^ pACYC184	This study
pZniA	pACYC184::ZniA – *K. pneumoniae* AJ218 *zniA* cloned into Chl^R^ pACYC184	This study
pET52b::*zniA*	*K. pneumoniae* AJ218 *zniA* cloned into Ap^R^ pET52b, with *pelB* signal peptide, C-terminal 10×His-tag, thrombin cleavage site.	This study

### Construction of *K. pneumoniae* gene deletion mutants

The *K. pneumoniae* Δ*znuA, ΔzniA*, and Δ*znuA* Δ*zniA* deletion strains were generated by adapting the gene gorging method (Wilksch et al., 2011). Briefly, NEBuilder HiFi DNA Assembly Master Mix (New England Biolabs, NEB) was used to flank a Km resistance (Km^R^) cassette from pKD4 with ~500 bp amplicons of sequence from upstream and downstream of the *znuA* or *zniA* genes. The linear construct (200 ng) was electroporated into electrocompetent *K. pneumoniae* containing the plasmid pACBSR, which encodes an L-arabinose-inducible lambda Red recombinase gene. Transformants were selected for acquisition of Km^R^ and correct insertions screened by PCR. The pACBSR plasmid was cured from successful transformants by passage in 0.2% (w/v) L-arabinose-supplemented Luria Bertani (LB) broth. The Km^R^ cassette was excised from deletion strains by pFLP-BSR, which induced recombination at the FRT sites. The pFLP-BSR plasmid was then cured by overnight passage in 0.2% (w/v) L-arabinose-supplemented LB. Complementation vectors were constructed by PCR amplification of pACYC184, and the *znuA* or *zniA* coding regions, and assembly with NEBuilder as per manufacturer’s protocols. The pZnuA complementation vector contains the *znuA* gene with its native promoter, inserted into the pACYC184 *tet* gene in the opposite orientation to the coding sequence. Due to the lack of a native promoter upstream of *zniA*, the pZniA complementation vector was cloned into the pACYC184 *tet* gene sequence in the same orientation thereby allowing constitutive expression from the *tet* promoter. Oligonucleotides for all constructs are listed in **Supplementary Table 2**.

### Elemental analyses

Elemental content analyses were performed by inductively coupled plasma-mass spectrometry using an Agilent 8900 ICP-QQQ-MS (Agilent Technologies) with established protocols (Neville et al., 2021; Brazel et al., 2022). For bacterial elemental analyses, samples were prepared as described previously (Maunders et al., 2022). Briefly, *K. pneumoniae* strains were subcultured in Zn(II)-limited or Zn(II)-supplemented media at 37°C with aeration to mid-log phase (OD_600 =_ 0.6-0.8) and harvested by centrifugation at 7,000 × *g* for 10 min at room temperature. The cell pellets were washed twice in phosphate buffered saline (PBS) containing 5 mM ethylenediaminetetraacetic acid, followed by two washes in PBS. Bacterial cell pellets were then desiccated overnight at 95°C, followed by digestion of organic material in 250 μL 65% (v/v) HNO_3_ at 95°C for 20 min. For media analyses, samples were diluted 1:2 with 65% (v/v) HNO_3_ and incubated at 95°C for 20 min. Digested media and whole cell pellet samples were then centrifuged at 18,000 × *g* for 25 min at room temperature and the supernatant diluted to a final concentration of 3.25% (v/v) HNO_3_ in MilliQ H_2_O (Neville et al., 2021; Brazel et al., 2022).

### RNA isolation and qRT-PCR analyses

*K. pneumoniae* strains were grown in Zn(II)-limited or Zn(II)-supplemented media at 37°C with aeration to mid-log phase (OD_600 =_ 0.6-0.8) and harvested by centrifugation at 18,000 × *g* for 5 min at room temperature. Cells were resuspended in RNA Protect Bacteria Reagent (Qiagen) and total RNA extracted using the RNeasy Mini Kit with two rounds of on-column DNase I digestion, as per manufacturer’s instructions, with final concentrations determined by spectrophotometry. Quantitative real-time-PCR (qRT-PCR) was performed on the QuantStudio 7 Real-Time PCR System using the SuperScript III Platinum SYBR Green One-Step qPCR Mix (ThermoFisher Scientific) using *rpoD* to normalize gene expression, essentially as described previously (Maunders et al., 2022). Oligonucleotides used are listed in **Supplementary Table 2**.

### ZniA expression and purification

The mature sequence of *zniA* was predicted by XtalPred (Slabinski et al., 2007). The sequence was PCR amplified from *K. pneumoniae* AJ218 genomic DNA and cloned into the pET52b vector with a *pelB* signal peptide for periplasmic secretion and a C-terminal decahistidine tag preceded by a thrombin cleavage site by restriction free cloning (van den Ent and Löwe, 2006) to form pET52b::*zniA*. *E. coli* BL21(DE3) pET52b::*zniA* was grown overnight in LB broth with aeration and sub-cultured the following day into 6 L. At mid-log phase (OD_600 =_ 0.6) cells were induced with 0.5 mM isopropyl β-D-1-thiogalactopyranoside, and cultures incubated 16 hours at 20°C with aeration. Cells were harvested by centrifugation at 5,000 rpm at 4°C for 25 min. The harvest cells were resuspended in 50 mM Tris.HCl pH 8.0, 300 mM NaCl, 10 mM imidazole. Cells were lysed by homogenization (Avestin EmulsiFlex-C3) and centrifuged at 4°C and 17,000 × g for 45 min. The supernatant was passed over Ni-NTA resin pre-equilibrated with wash buffer [50 mM 4-(2-hydroxyethyl)-1-piperazineethanesulfonic acid (HEPES) pH 8.0, 4 mM imidazole, 500 mM NaCl], washed with 1 column volumes of wash buffer, and eluted in 50 mM HEPES pH 8.0, 300 mM imidazole, 500 mM NaCl. Fractions containing ZniA were pooled, digested with 100 units of thrombin (Sigma-Aldrich, USA), and dialyzed overnight in 25 mM HEPES pH 8.0, 150 mM NaCl. Dialyzed ZniA was combined with 15 μL aminobenzamadine conjugated to agarose beads (Sigma-Aldrich, USA) to remove thrombin via gentle agitation at 4°C for 60 min. The sample was centrifuged at 500 × *g*, 4°C for 5 min to separate the agarose beads and ZniA-containing supernatant. The protein was then concentrated using a 10 kDa molecular weight cut off centrifugal filter (Millipore, USA). ZniA was then resolved by size exclusion chromatography using a Superdex S200 increase column (GE Healthcare, USA) in 25 mM HEPES pH 8.0, 150 mM NaCl.

### Crystallization, diffraction data collection, and structure building

Recombinant, tag-cleaved ZniA was concentrated to 40 mg.mL^-1^ and crystallized in 0.2 M ammonium sulfate with 23% (w/v) PEG 4000. Crystals were looped and cryoprotected in 0.2 M ammonium sulfate with 23% (w/v) PEG 4000 containing 15% (w/v) glycerol. Crystals were sent to the National Synchrotron Light Source at Brookhaven National Laboratory in Upton, New York for screening and data collection/A single crystals diffracted to a resolution of 1.59 Å in space group C222_1_ was selected for further processing. Phases were retrieved using molecular replacement Phaser MR in Phenix (Adams et al., 2011) using an AlphaFold2 (Jumper et al., 2021) generated model. Manual refinements to the model were made using Coot (Emsley et al., 2010) and further refinement was performed with Phenix. PyMol (Version 1.2r3pre, Schrödinger, LLC) was used for molecular modeling, graphics, and electrostatic surface mapping. Structure coordinates were deposited to the Protein Data Bank with the accession number 8SVC.

### Phase contrast microscopy and cell morphology analysis

*K. pneumoniae* AJ218 strains were grown on Zn(II)-limited or Zn(II)-supplemented agar plates for microscopic analyses. For morphological analysis, single colonies were smeared onto glass slides, fixed with 96% (v/v) methanol and stained with 0.1% (v/v) crystal violet. Phase contrast microscopy was carried out with a Leica DMI4000 B microscope equipped with a HCX PL Fluotar 100 ×/1.30 oil immersion objective lens. Images were acquired with the Leica Application Suite (LAS) v4.6. and analyzed using Fiji (v2.3.0/1.53q) (Schindelin et al., 2012). Cells were detected using the medial axis method in the Fiji plugin MicrobeJ (v5.13n) (Ducret et al., 2016) using the following threshold settings: area (0.2-2.5 µm^2^), length (0.5-3.5 µm), width (0-1.2 µm), and curvature (0-max). All images were manually inspected to ensure that cells had been correctly detected. To investigate cell shape, the curvature of 100 randomly selected cells was determined by MicrobeJ as the ‘reciprocal of the radius of curvature measured between the endpoints and the center of the medial axis of the cell’. Using Fiji software, representative images were cropped, minimally processed using the sharpen tool, and a 5 µm scale bar added.

### Scanning electron microscopy

*K. pneumoniae* AJ218 strains were grown on Zn(II)-limited or Zn(II)-supplemented agar plates overnight. Bacteria were then harvested, resuspended in PBS, and applied to poly-L-lysine treated coverslips and incubated at room temperature for 30 min to enable adherence. Coverslips with adhered cells were fixed in 2.5% (v/v) glutaraldehyde, incubated for 60 min at room temperature, then washed twice with PBS. Coverslips were then post-fixed in 1% (w/v) osmium tetroxide for 60 min, washed twice in ddH_2_O and dehydrated through a series of 10 min incubations in increasing ethanol concentrations (incrementing by 10% ethanol each time), and several final 30 min incubations in 100% ethanol. Coverslips were dried in a Leica EM CPD 300 critical point dryer, mounted on scanning electron microscopy stubs, and coated in gold, using a Quorum QT5000 sputter coater. Stubs were imaged by a Hitachi SU7000 Field Emission Scanning Electron Microscope at 5 kV.

### Chemical stress assays

The salt stress assay used overnight cultures of each *K. pneumoniae* AJ218 strain, resuspended to a final OD_600_ of 0.05, in Zn(II)-limited or Zn(II)-supplemented media that also contained 0 mM, 600 mM, or 1 M NaCl. The cultures were grown in 96-well microtitre plates statically for 24 h at 37°C. At 24 h, the final OD_600_ of each culture was measured. For the paraquat stress assays, overnight cultures of each *K. pneumoniae* AJ218 strain were harvested and resuspended to final OD_600_ of 1.0, and then inoculated at 1:100 into molten Zn(II)-limited agar. Inoculated agar cultures were then poured into sterile petri dishes (Technoplas) and air-dried. Filter paper discs (6 mm diameter, Whatman) impregnated with 5 µL of 0 mM or 20 mM paraquat were then placed onto the *K. pneumoniae* plates and incubated overnight at 37°C. After incubation, zones of clearance were measured. Each stress assay was performed with three independent replicates.

### Animal infection experiments

Five-week-old female BALB/c mice were anaesthetized with isofluorane, and intranasally infected with 1.5-2.5 × 10^6^ colony forming units (CFUs) of *K. pneumoniae* B5055 wild type or derivative strain prepared in nutrient broth (Media Preparation Unit, University of Melbourne) supplemented with 10% (v/v) serum broth [heat inactivated horse serum (New Zealand origin, Thermo Fisher Scientific)]. Post-infection, mice were monitored for 36 h for clinical signs of infection, specifically related to weight loss, respirations, appearance, and behavior/activity impairment, in accordance with University of Melbourne Animal Ethics Committee approved guidelines (Animal Care and Use Standards, and approved Project #20018), and the Australian Code for the Care and Use of Animals for Scientific Purposes. At 36 h post-infection, mice were humanely euthanized by CO_2_ asphyxiation, weighed, followed by harvesting of murine tissues (nasopharynx, lungs, blood, and spleen) and lavage of the pleural cavity. Tissues were then processed for enumeration of bacterial CFU by homogenization (Precellys homogeniser), serial dilution and plating on Cm-supplemented LB agar. Data represent the geometric mean ± standard deviation (SD) of two independent experiments (5 mice per group) with each point representing data from an individual mouse.

### Statistical analyses

Unless otherwise stated, data represent the mean ± standard error of the mean (SEM) from three independent experiments. Statistical analyses were performed in GraphPad Prism (Version 10.0.1), with test details described. For the murine infection experiments, a one-way ANOVA assuming nonparametric, non-matched data, corrected for multiple comparisons using statistical hypothesis testing (Kruskal-Wallis test with Dunn’s multiple comparisons test) was used.

## Data availability statement

The datasets presented in this study can be found in online repositories. The names of the repository/repositories and accession number(s) can be found below: http://www.wwpdb.org/, 8SVC.

## Ethics statement

The animal study was approved by University of Melbourne Animal Ethics Committee. The study was conducted in accordance with the local legislation and institutional requirements.

## Author contributions

EM: Conceptualization, Formal Analysis, Investigation, Methodology, Validation, Writing – original draft, Writing – review & editing. MG: Data curation, Formal Analysis, Investigation, Methodology, Writing – review & editing, Writing – original draft. KG: Investigation, Writing – review & editing. BC: Investigation, Writing – review & editing. VB-W: Investigation, Methodology, Writing – review & editing. GC: Methodology, Writing – review & editing. DN: Writing – review & editing, Methodology. CL: Writing – review & editing, Methodology. SN: Methodology, Writing – review & editing. TM: Conceptualization, Formal Analysis, Methodology, Funding acquisition, Supervision, Writing – review & editing. CM: Conceptualization, Formal Analysis, Methodology, Funding acquisition, Project administration, Supervision, Writing – original draft, Writing – review & editing. AT: Conceptualization, Formal Analysis, Investigation, Methodology, Supervision, Validation, Writing – original draft, Writing – review & editing.
