# Supplementary material for: Zinc acquisition and its contribution to Klebsiella pneumoniae virulence
Source: Front Cell Infect Microbiol. 2024 Jan 5;13:1322973. doi: 10.3389/fcimb.2023.1322973 (PMC10797113; doi:10.3389/fcimb.2023.1322973)
Supplement: Supplementary file 1 [file DataSheet_1.docx]

Supplementary Material

**Supplementary Table 2. Oligonucleotide primers used in this study.**

**Supplementary Figure 1. Conservation analyses of the *znuCBA-mepM* and *zniCBA* loci.**

**Supplementary Figure 2. *K. pneumoniae* growth in Zn(II)-supplemented media and elemental content analyses.**

**Supplementary Figure 3. Morphological variation in *K. pneumoniae* complement strains.**

**Supplementary Figure 4. Characterization of the *K. pneumoniae* B5055 derivative strains.**

**Supplementary Table 2. Oligonucleotides used in this study.**

| **Name** | **Sequence (5’ → 3’)** | **Purpose** |
| --- | --- | --- |
| pKD4F | tgtgtaggctggagctgcttc | Gene deletion: Amplification of KanR cassette |
| pKD4R_ex | catatgaatatcctccttagttcctattcc |  |
| znuA_KO_LF_F | ggcggccgcgggaattcgatGGTTCATCCAGCACCAGC | Gene deletion: *znuA* left flank amplification |
| znuA_KO_LF_R | aagcagctccagcctacacaCATCGAAATAAGTGAGTGTGATG |  |
| znuA_KO_RF_F | ctaaggaggatattcatatgCGAGGAAGTGAATACGTGAAC | Gene deletion: *znuA* right flank amplification |
| znuA_KO_RF_R | gccgcgaattcactagtgatGTTATCCACACGGGTATAGGTG |  |
| znuA_LF_chk_F | TACAATTCGCCCCTGAAGGTC | Gene deletion: Confirmation of *znuA* gene deletion |
| znuA_RF_chk_R | AACTGGCTTTGCTCACGTTTG |  |
| zniA_KO_LF_F | CCTCCTGCATCTGCTGTTTG | Gene deletion: *zniA* left flank amplification |
| zniA_KO_LF_R | agcctacacaCATTTCCTTACCCCATCAATGTG |  |
| zniA_KO_RF_F | tattcatatgCAGGTTGTCTGGTCGTCC | Gene deletion: *zniA* right flank amplification |
| zniA_KO_RF_R | TCTCCGGCAAAGCATTCC |  |
| zniA_LF_chk_F | ccgccaacgctttctgtttg | Gene deletion: Confirmation of *zniA* gene deletion |
| zniA_RF_chk_R | gattttatctgggctcgctgg |  |
| pACYC184_F | ACGGGTGCGCATAGAAATTGC | Complementation: pACYC184 amplification for gene expression from native promoter |
| pACYC184_F2 | CGGTGCCTGACTGCGTTAG | Complementation: pACYC184 amplification for constitutive gene expression |
| pACYC184_R | GCGAGAAGCAGGCCATTATCG | Complementation: pACYC184 amplification |
| znuA_comp_F | gataatggcctgcttctcgcACGCTTGATAACACCCTCTG | Complementation: *znuA* amplification |
| znuA_comp_R | caatttctatgcgcacccgtTGCACGTATTCACTTCCTCG |  |
| zniA_comp_F | gctaacgcagtcaggcaccgATGAAACGTAGTGCAATAG | Complementation: *zniA* amplification |
| zniA_comp_R | gataatggcctgcttctcgcTTACTTCATGCTGTTAGC |  |
| pACYC_seq_F | GCTAACGCAGTCAGGCACC | Sequencing primer for pACYC184 inserts |
| pACYC_seq_R | CAGCAAGACGTAGCCCAGC |  |
| rpoD562F | gaagagatggatgacgacgaagacg | Real time qPCR: housekeeping control [61] |
| rpoD677R | gtacgcagctcggcgaatttctcac |  |
| znuA_rt_F | CGAGACCCAGGTTCTGCTG | Real time qPCR: *znuA* |
| znuA_rt_R | CACACCACTAAGTCTGCGTTC |  |
| znuB_rt_F | GGTTATCGCCGTGACCCTG | Real time qPCR: *znuB* |
| znuB_rt_R | GTGCGCCATGATGCCCAG |  |
| znuC_rt_F | CTACCAGCCCGAGAACCAC | Real time qPCR: *znuC* |
| znuC_rt_R | GACATCTCGCTGGCTCTCAC |  |
| zniA_rt_F | CGAAAACGCTGAATGTGGTAAGC | Real time qPCR: *zniA* |
| zniA_rt_R | GAGACGGTTCGAAGGTGTGC |  |
| zniC_rt_F | TTTCTTCCGCCGGTCAGTG | Real time qPCR: *zniC* |
| zniC_rt_R | ACATCCTGCACGTTGAGAGG |  |
| zinT_rt_F | GTTAACCGAAGTGGAGCGTC | Real time qPCR: *zinT* |
| zinT_rt_R | CGAGATCGCCATTGAGCAG |  |
| zupT_rt_F | GGTAGCGCAACCTTCATTGG | Real time qPCR: *zupT* |
| zupT_rt_R | CAGGCAGCATTTCCATCAACG |  |
| pitA_rt_F | CGAAGCGATTAACGGTTTCC | Real time qPCR: *pitA* |
| pitA_rt_R | GAACGCCGAAGAAGTTGAAG |  |
| mntH_rt_F | TATCGATCCGGGTAATTTCG | Real time qPCR: *mntH* |
| mntH_rt_R | ATCAGGTTAGCCCACACCAC |  |
| sitA_rt_F | GACGTGAGCTCCATTACTAAACC | Real time qPCR: *sitA* |
| sitA_rt_R | CGTTCGAGATTAAGCCCATTGC |  |
| mepM_rt_F | TCAGGCATTCAACAATCTGC | Real time qPCR: *mepM* |
| mepM_rt_R | TTCCGGGTGGTAAATGTAGG |  |
| F-RF-pelBZniA | GCTGCCCAGCCGGCGATGGCCATGGCAAAAACGCTGAATGTGGTAAGC | Cloning: Sub-cloning of *zniA* for expression vector |
| R-RF-p52ZniA | ActaccgcgtggcaccagagcgagctcCTTCATGCTGTTAGCGATAAGCTC |  |

**SUPPLEMENTARY FIGURE 1**

**
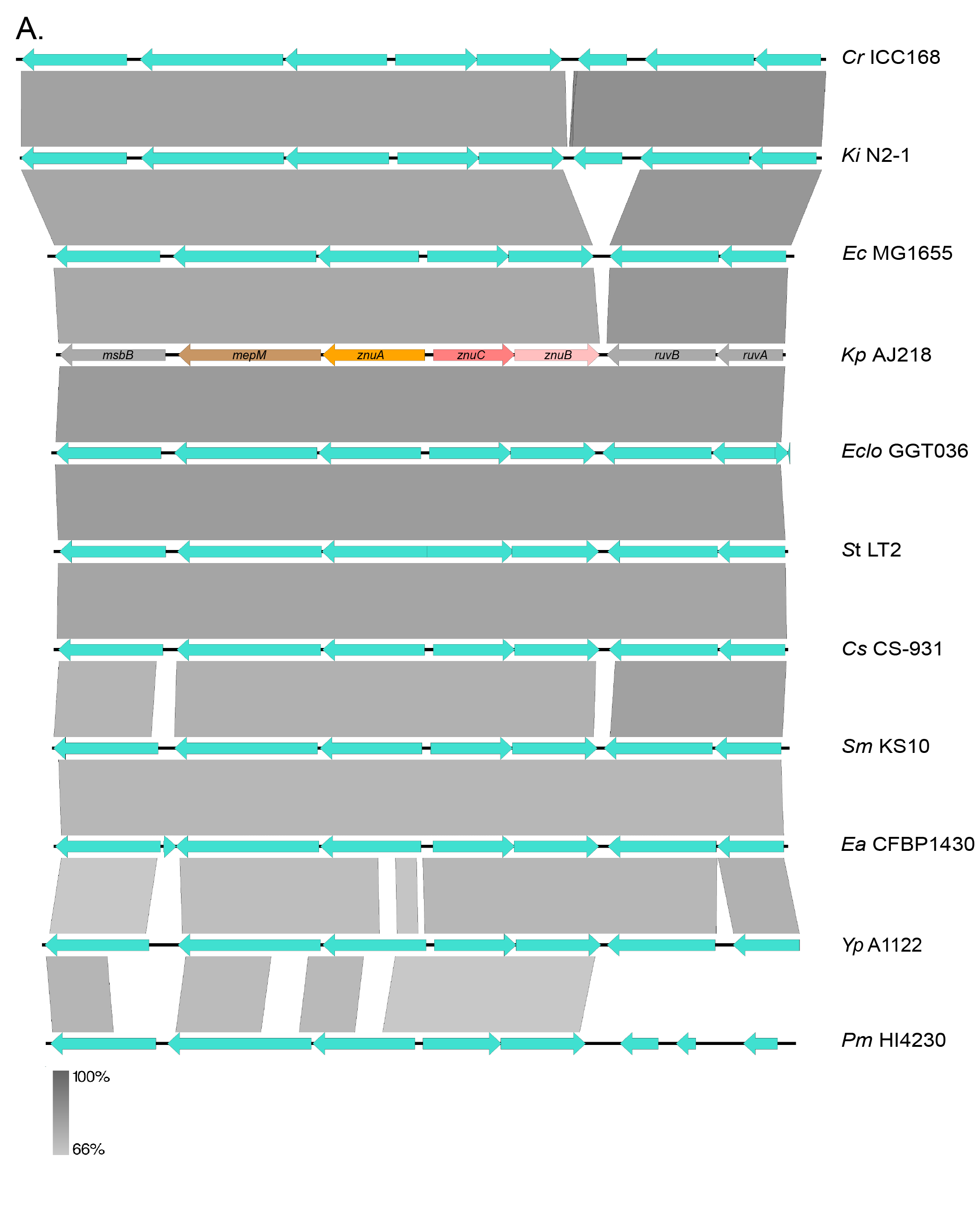
**

**
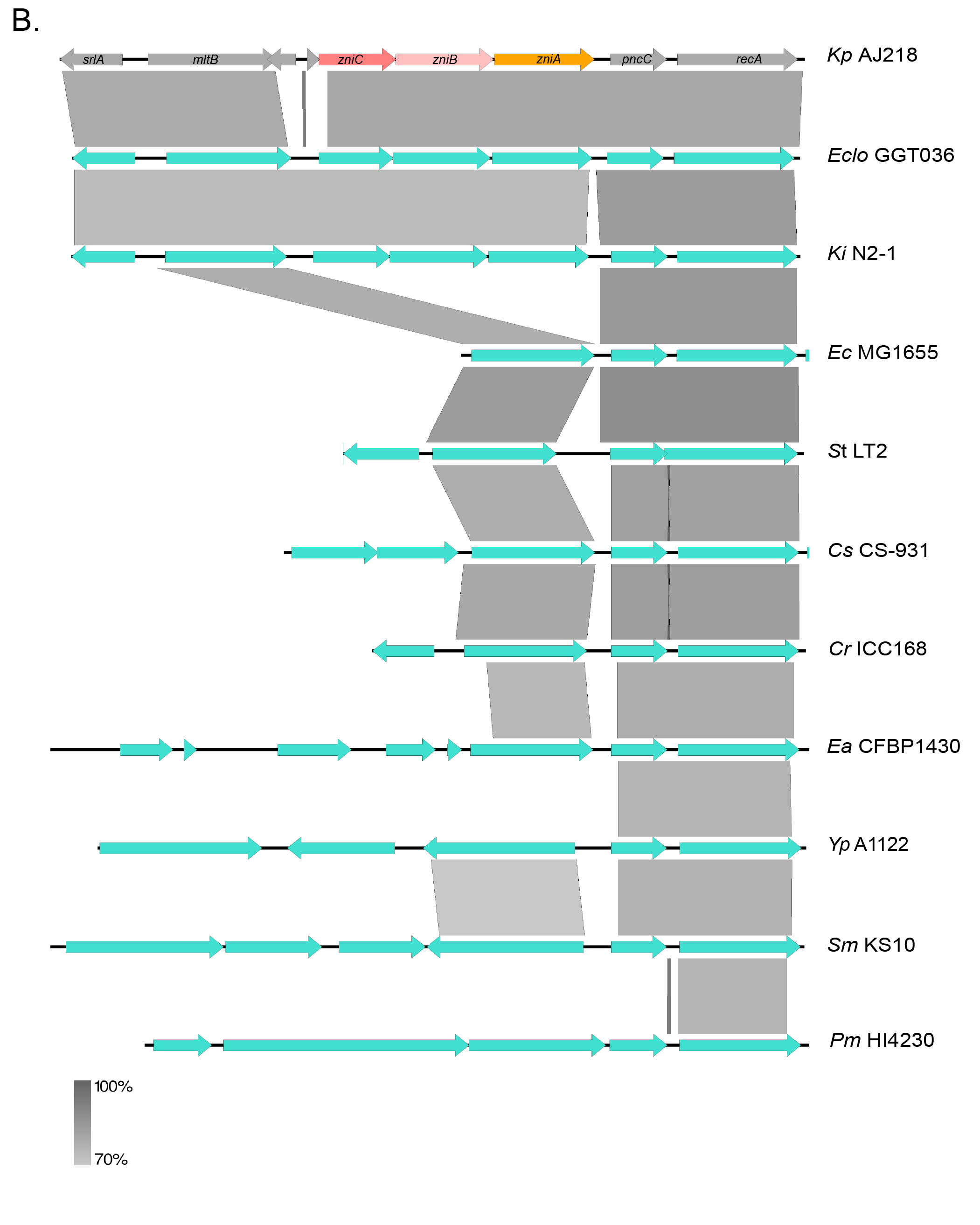
Supplementary Figure 1. Conservation analysis of the *znuCBA-mepM* and *zniCBA* loci.** Local genomic comparisons of the respective gene regions in the *K. pneumoniae* (Kp) AJ218 genome (accession NZ_LR130541), *Citrobacter rodentium* (Cr) ICC168 (NC_013716), *Cronobacter sakazakii* (Cs) CS-931 (CP027107), *Enterobacter cloacae* (Eclo) GGT036 (CP009756), *Erwinia. amylovora* (Ea) CFBP1430 (FN434113), *E. coli* (Ec) MG1655 (U00096), *Kluyvera intermedia* (Ki) N2-1 (P045845), *P. mirabilis* (Pm) HI4320 (NC_010554), *S. enterica* servar Typhimurium (St) LT2 (GCF_000006945.2), *Serratia marcescens* (Sm) KS10 (NZ_CP0277798), and *Yersinia pestis* (Yp) A1122 (NC_017168) genomes for the (**A**) *znuCBA-mepM* and (**B**) *zniCBA* loci. Genes are indicated by colored arrows with *K. pneumoniae* nomenclature used. Where applicable, levels of nucleotide homology between adjacent strains are indicated by the grey shading between loci, with scale bar shown.

**SUPPLEMENTARY FIGURE 2**

**
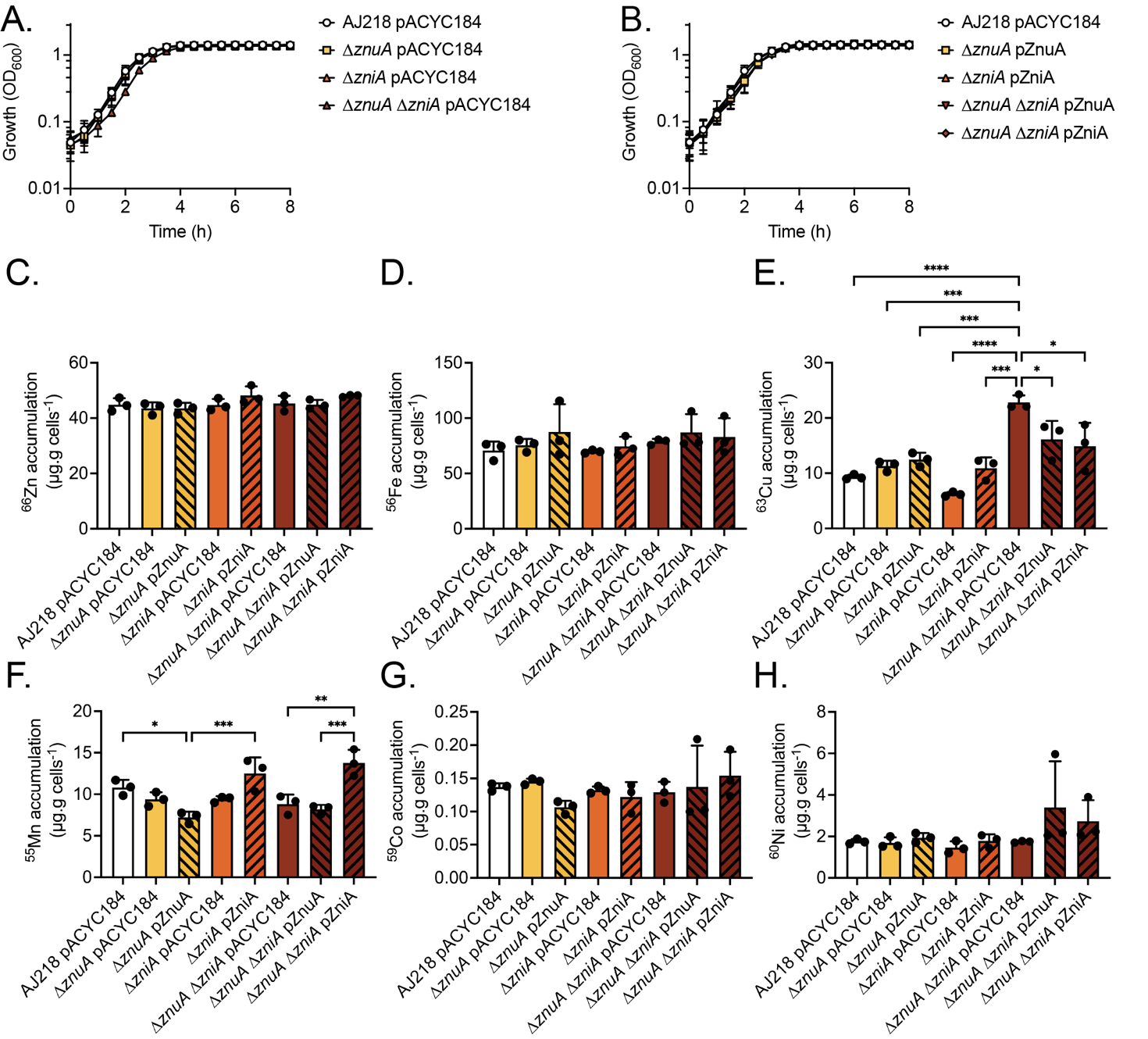
Supplementary Figure 2. *K. pneumoniae* growth in Zn(II)-supplemented media and elemental content analyses.** *K. pneumoniae* AJ218 pACYC184, mutant (**A**), and complemented strains (**B**) growth phenotypes in Zn(II)-supplemented media. Data represent mean OD_600_ values (± SEM) from three independent experiments. Whole cell accumulation of ^66^Zn (**C**), ^56^Fe (**D**), ^63^Cu (**E**), ^55^Mn (**F**), ^59^Co (**G**), and ^60^Ni (**H**) for the wild type and derivative strains grown in Zn(II)-supplemented media. Data represent mean (± SEM) μg metal.g cells^-1^ (dry weight) from at least three independent experiments. Statistical significance of differences was determined by one-way ANOVA analysis with Bonferroni post-test; ns, not significant; *, *P* < 0.05; **, *P* < 0.01; ***, *P* < 0.001; ****, *P* < 0.0001.

**SUPPLEMENTARY FIGURE 3**

**
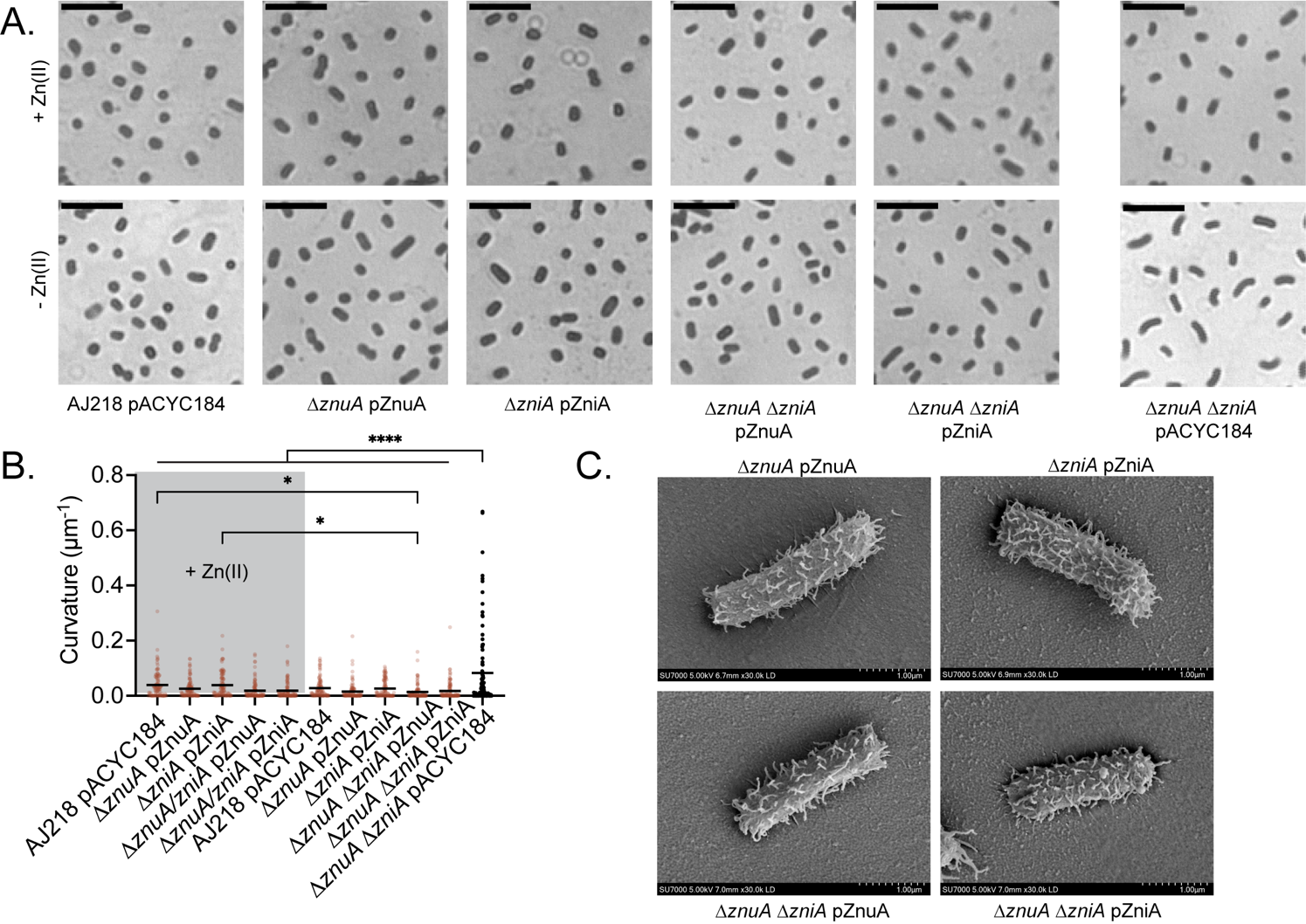
**

**Supplementary Figure 3. Morphological variation in *K. pneumoniae* complement strains.** (**A**) Representative images of *K. pneumoniae* AJ218 wild type and complement strains grown in Zn(II)-limited media in the presence or absence of 10 µM ZnSO_4_, stained with crystal violet. Scale bar (5 µM) is shown. (**B**) Curvature analyses of the strains using Fiji plugin MicrobeJ. Statistical significance of differences was determined by one-way ANOVA analysis with Tukey post-test. ns, not significant; *, *P* < 0.05; **, *P* < 0.01; ***, *P* < 0.001; ****, *P* < 0.0001. (**C**) Representative images of the wild type and derivative strains grown in Zn(II)-limited media visualized at 5 kV by field emission scanning electron microscopy. Scale bars are shown.

**SUPPLEMENTARY FIGURE 4**

**
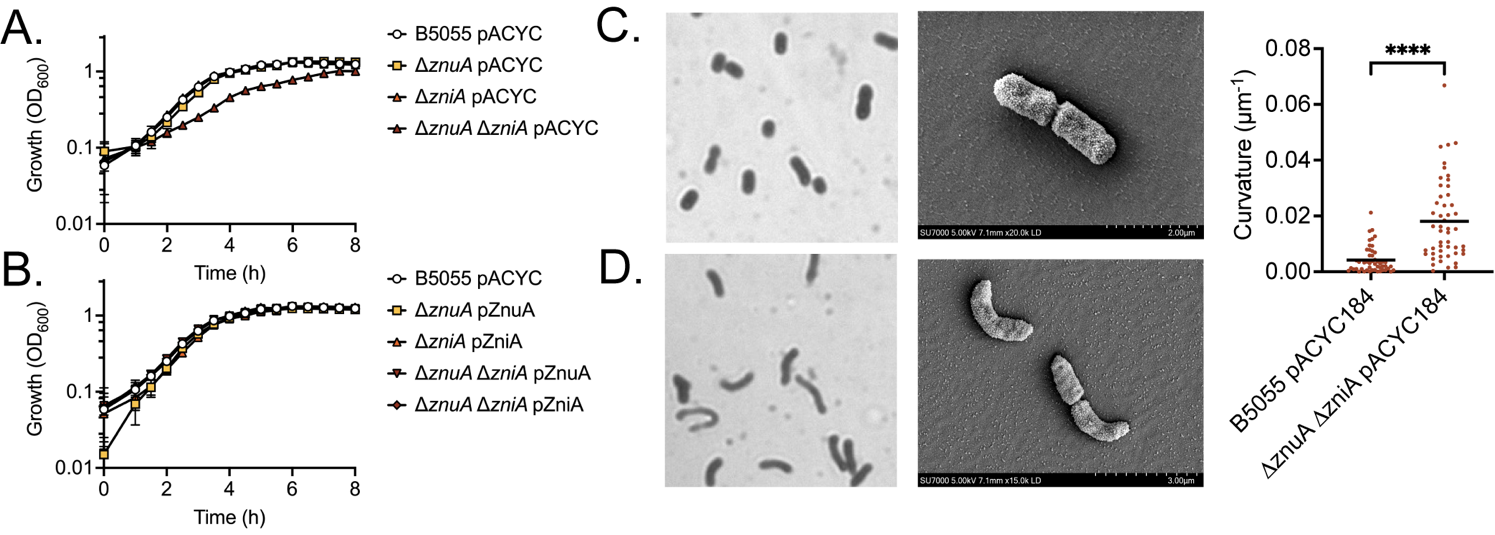
**

**Supplementary Figure 4. Characterization of the *K. pneumoniae* B5055 strains.** *K. pneumoniae* B5055 pACYC184, mutant (**A**), and complemented strains (**B**) growth phenotypes in Zn(II)-limited media. Data represent mean OD_600_ values (± SEM) from three independent experiments. Representative images of *K. pneumoniae* B5055 (**C**) and the ∆*znuA* ∆*zniA* strain (**D**) grown in Zn(II)-limited media, stained with crystal violet (left), visualized at 5 kV by field emission scanning electron microscopy with scale bars shown (middle) and with curvature analyses of the strains using Fiji plugin MicrobeJ (right). Statistical significance of differences was determined by one-way ANOVA analysis with Tukey post-test. ns, not significant; *, *P* < 0.05; **, *P* < 0.01; ***, *P* < 0.001; ****, *P* < 0.0001..
